# Incorporation of broccoli leaf-derived CDs and Ag/AgCl nanoparticles into PVA/chitosan as multifunctional food packaging films

**DOI:** 10.1039/d6ra04270h

**Published:** 2026-07-29

**Authors:** Nhung Thi Tran, Le Minh Nguyen, Huynh-Anh Le, Thanh-Nhan Le

**Affiliations:** a Ho Chi Minh City University of Technology and Engineering 01 Vo Van Ngan Street, Thu Duc Ward Ho Chi Minh City 700000 Vietnam nhungtt@hcmute.edu.vn

## Abstract

The incorporation of hybrid nanofillers with complementary functionalities into packaging materials has emerged as an effective strategy for achieving multifunctionality. In this study, multifunctional PVA/chitosan-based films were fabricated by simultaneously incorporating broccoli leaf-derived N,S-doped carbon dots (CDs) and *in situ* synthesized Ag/AgCl nanoparticles (0–0.70% Ag relative to polymer mass). CDs served as reducing agents for the formation of ∼27.4 nm Ag/AgCl nanoparticles within the polymer matrix. The resulting films exhibited tunable mechanical and functional properties depending on the Ag content. At 0.45% Ag loading, the composite film displayed enhanced mechanical properties (tensile strength of 61.1 MPa and elongation at break of 190%) and excellent UV-shielding (∼93% UVA and nearly 100% UVB/UVC blocking). The films also showed strong antioxidant activity (∼93% 2,2-diphenyl-1-picrylhydrazyl scavenging), mainly attributed to CDs. Furthermore, the films demonstrated significant antibacterial activity against *Escherichia coli* and *Staphylococcus aureus*, primarily attributed to Ag/AgCl nanoparticles, with inhibition zones of 13.4 and 12.9 mm, respectively. Practical tests confirmed that the composite film significantly extended the shelf life of guavas and strawberries, outperforming commercial polyethylene films. These results highlight its strong potential for multifunctional food packaging applications.

## Abstract 2

*In situ* synthesized Ag/AgCl nanoparticles and broccoli-derived N-doped CDs were incorporated into PVA/chitosan to develop a multifunctional packaging film with enhanced UV-blocking, antibacterial, and antioxidant properties.

## Graphical abstract

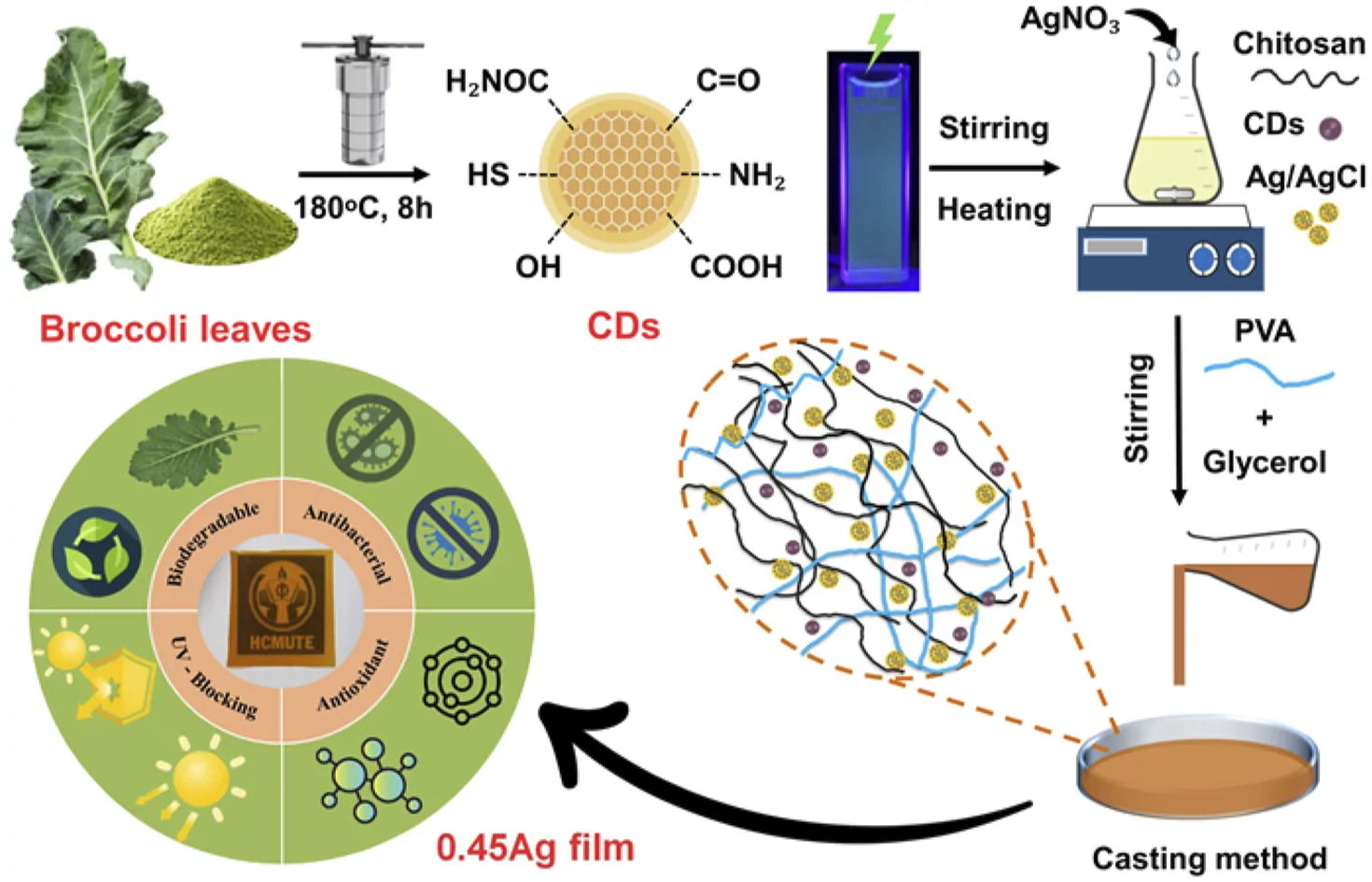

## 1 Introduction

Conventional packaging materials, predominantly derived from petroleum-based plastics such as polyethylene (PE), polypropylene (PP), polystyrene (PS), and polyethylene terephthalate (PET), primarily function as passive barriers and lack active functionalities to prevent microbial contamination, oxidative degradation, and ultraviolet (UV) radiation damage, which are key factors responsible for food spoilage.^1^ Moreover, growing concerns regarding the environmental sustainability and ecological impact of these non-biodegradable plastics have accelerated the shift toward developing biodegradable packaging systems that integrate multiple protective functions to enhance food preservation.^2^

Biodegradable polymers with film-forming capabilities, particularly polysaccharides,^3–5^ proteins,^6^ poly(lactic acid),^7^ poly(propylene carbonate),^8^ and poly(hydroxybutyrate-*co*-hydroxyvalerate) (PHBV)^9^ offer promising alternatives. Among them, chitosan, obtained by the deacetylation of chitin sourced from shrimp and crab shells, is a widely utilized material in biodegradable packaging films, owing to its strong mechanical properties, effective barrier performance, and inherent antimicrobial activity.^3^ Nevertheless, further improvements in thermal stability, mechanical robustness, and moisture resistance are required, along with addressing technical challenges that limit its cost-effective large-scale production.^10^ Polyvinyl alcohol (PVA), a biodegradable thermoplastic polymer synthesized *via* polymerization of vinyl acetate followed by hydrolysis, is considered non-toxic and exhibits excellent oxygen barrier, film-forming ability, and resistance to acids and alkalis.^1,3,11^ Moreover, PVA exhibits good compatibility with chitosan, which is attributed to the formation of a dense intermolecular network through hydrogen bonding, van der Waals forces, and electrostatic interactions between the protonated amine groups of chitosan and the hydroxyl groups of PVA. Therefore, blending chitosan with PVA has attracted significant research interest due to its ability to improve mechanical and barrier properties, while enabling industrial-scale film fabrication through techniques such as injection and blown molding, owing to the thermoplastic nature of PVA.^3,11^

To achieve multifunctional packaging, increasing research efforts have focused on incorporating various nanofillers into PVA/chitosan-based films, including plant extracts, essential oils, metallic and metal oxide nanoparticles, and carbon-based nanomaterials such as graphene, graphene oxide, and carbon dots (CDs).^11–14^ Notably, CDs have attracted significant attention due to their distinct optical properties, biocompatibility, and environmental safety, positioning them as a promising innovation in packaging systems.^2^ Their incorporation provides strong antioxidant activity and UV-blocking capability without compromising transparency or mechanical strength.^15,16^ For instance, Zhao *et al.* fabricated CDs from banana paste and incorporated them into PVA films, achieving excellent UV shielding of 98.5%, a 2,2-diphenyl-1-picrylhydrazyl radical (DPPH) free radical scavenging activity of 72.81%, and antibacterial activity against *Escherichia coli* (*E. coli*) and *Staphylococcus aureus* (*S. aureus*), with inhibition zones of 9.52 mm and 9.05 mm, respectively.^17^ Furthermore, CDs can be synthesized from a wide range of carbon-rich precursors, including biomass, making them promising candidates for sustainable material development.^18^ However, the intrinsic antibacterial activity of CDs is generally limited. Heteroatom doping, particularly with nitrogen, helps enhance their antibacterial performance by improving interactions with bacterial cell membranes. In contrast, silver-based nanomaterials are well known for their broad-spectrum antibacterial activity and have been widely incorporated into polymer films to impart strong antimicrobial properties.^19–21^ For example, Yang *et al.* demonstrated that silver nanoparticle-incorporated PVA/chitosan films exhibit enhanced mechanical strength, improved water resistance, and significant antibacterial activity against *E. coli*, with an inhibition zone of 16.07 mm.^22^ However, the properties of silver nanostructures strongly depend on the synthesis conditions. Generally, strong reducing agents yield silver nanoparticles with small sizes, while mild reducing agents, especially plant-derived extracts, tend to promote the formation of larger particles or Ag/AgCl nanostructures.^23,24^ For instance, by using plant extracts from the leaves of *I. jinicuil*, and *H. rovirosae* (Hr), along with the aerial parts of *A. arvensis* as dual reducing and stabilizing agents, Torres López *et al.* successfully synthesized Ag/AgCl nanoparticles with enhanced bactericidal activity.^25^ Additionally, Ag/AgCl nanoparticles were also synthesized under Ultraviolet C (UVC) irradiation and subsequently incorporated into PVA films for meat packaging applications.^26^ Notably, the resulting films exhibited a low degree of silver migration, which was attributed to the low solubility of AgCl, thereby reducing the risk of food contamination. Recently, the design of hybrid packaging systems incorporating various nanofillers to achieve multifunctionality has attracted increasing research attention.^27,28^ For example, Ananthi *et al.* reported the incorporation of cow milk-derived CDs and UV-assisted synthesized silver nanoparticles into agar-based packaging films, in which silver nanoparticles were introduced to enhance the antibacterial activity of the CDs.^29^ Similarly, Du *et al.* synthesized Ag@CDs nanocomposites, where Ag nanoparticles were pre-formed using CDs derived from the pyrolysis of PEI and histidine as reducing agents and subsequently incorporated into PVA/chitosan films.^27^ At a loading of 3 wt%, the films exhibited inhibition zones of 13.8 mm and 12.8 mm against *E. coli* and *S. aureus*, respectively.

In this study, a multifunctional packaging film based on a PVA/chitosan blend was fabricated *via* an *in situ* approach by simultaneously incorporating broccoli leaf-derived CDs and Ag/AgCl nanoparticles. Owing to the presence of abundant functional groups and N,S-heteroatom doping, the CDs acted as reducing agents, while chitosan served as a stabilizing agent, enabling the formation of Ag/AgCl nanoparticles without the need for purification steps or the introduction of potentially toxic substances into the film-forming system. The green, *in situ* synthesis of Ag nanoparticles using fruit and plant extracts as reducing agents within a PVA/chitosan network for packaging films has recently also been explored by Gasti *et al.* and Hunashyal *et al.*^30,31^ To the best of our knowledge, the simultaneous and *in situ* incorporation of CDs and Ag/AgCl nanoparticles into a PVA/chitosan blend for the development of multifunctional packaging films has not yet been reported. The effects of CDs and silver contents (0–0.70% relative to the polymer mass) on mechanical properties, UV-blocking performance, water resistance, antioxidant activity, and antibacterial efficacy against *E. coli* and *S. aureus* were systematically investigated. The results demonstrate that the resulting composite films exhibit significantly improved mechanical properties, strong UV-shielding capability, and excellent free radical scavenging activity compared to the pristine polymer films. The practical applicability of the composite films was validated through preservation studies on UV-exposed guavas and strawberries, where they exhibited superior performance relative to commercial polyethylene (PE) films, thereby underscoring their strong potential for sustainable food packaging applications.

## 2 Experiments

### 2.1 Chemicals

Glycerol (C_3_H_8_O_3_, 99%), acetic acid (CH_3_COOH, 96.4%), sulfuric acid (H_2_SO_4_, 95–98%), methanol (CH_3_OH, 99.7%), hydrochloric acid (HCl, 36–38%), and peptone were purchased from XiLong Scientific Co., Ltd, China. Nutrient Broth and polyvinyl alcohol (PVA, *M*_w_ = 65 000–115 000 Da) were supplied by HiMedia Laboratories Pvt. Ltd, India. 2,2-Diphenyl-1-picrylhydrazyl (DPPH, >97.0%) was obtained from Tokyo Chemical Industry (TCI), Japan. Chitosan (>75% of deacetylation) was purchased from a local manufacturer in Vietnam. The broccoli leaf-derived CDs dispersion (25 mg mL^−1^) was synthesized *via* a hydrothermal process at 180 °C for 8 h using dried broccoli leaf powder as the carbon source, following our previous report.

### 2.2 *In situ* fabrication of CDs/Ag/AgCl nanocomposites incorporated into PVA/chitosan film

In a typical procedure, 75 mL of a 2 wt% chitosan solution (prepared in 1.0 v/v% acetic acid) and 10 mL of broccoli leaf-derived carbon dots (CDs) solution (25 mg mL^−1^) were added to an Erlenmeyer flask, and the mixture was gently stirred for 3 h. The solution was then heated to 80 °C and maintained at this temperature for 30 min to ensure complete homogeneity. Subsequently, an aliquot of AgNO_3_ solution (0.1 M) was added, and the mixture was continuously stirred for an additional 2 h. During the reaction, the solution color gradually changed from yellowish-brown to reddish-brown, indicating the formation of Ag/AgCl nanoparticles.

For film fabrication, 25 mL of a 14 wt% PVA solution, prepared by dissolving PVA in distilled water at 90 °C with stirring for 2 h, was added to the previously prepared Ag/AgCl dispersion and stirred for an additional 3 h. Subsequently, 0.8 mL of 10 wt% glycerol was added as a plasticizer, and the mixture was further stirred for 1 h. The resulting film-forming solution was cast into Petri dishes of 9 cm in diameter and allowed to dry at room temperature for 48 h. The dried films were then peeled off and stored in a desiccator for at least 48 h prior to characterization.

The volume of 0.1 M AgNO_3_ solution was systematically varied (0.70–3.25 mL) to achieve Ag contents ranging from 0.15 to 0.70% relative to the total weight of PVA and chitosan. The resulting films were labeled according to the Ag content. The pristine PVA/chitosan film (denoted as PC) and the PC film loaded with CDs (PC/CDs) were prepared under the same conditions to serve as control samples.

### 2.3 Characterization

#### 2.3.1 Characterization of CDs/Ag/AgCl nanocomposites

The optical properties of the synthesized Ag/AgCl nanocomposites were characterized by UV-vis spectroscopy (UH3500, Hitachi, Japan). Their morphology was examined using transmission electron microscopy (TEM, JEM-2100, JEOL, Japan), operated at an accelerating voltage of 80 kV. The crystal structure of the resulting Ag/AgCl nanoparticles was analyzed using an X-ray diffractometer (XRD, EMPYREAN, Malvern Panalytical, UK), operated over a scanning range of 5–80° with Cu Kα radiation (*λ* = 1.540 598 Å), while their chemical composition was characterized using a Fourier transform infrared (FTIR) spectrometer (FT/IR-4700, JASCO, Japan).

#### 2.3.2 Film characterization

##### 2.3.2.1 Thickness

The thickness of each investigated film was determined using a digital micrometer (SHAHE, 0–25 mm, ± 1 µm, China). The measurements were repeated at 3 random positions and the results were reported as mean ± standard deviation (SD).

##### 2.3.2.2 Optical properties

All investigated films were cut into strips with dimensions of 10 × 50 mm^2^. Their optical transmittance was measured over the wavelength range of 190–1000 nm using a UV-vis spectrophotometer (UH-5300, Hitachi, Japan), with air serving as the reference. The film opacity was calculated using the following equation:^32^1
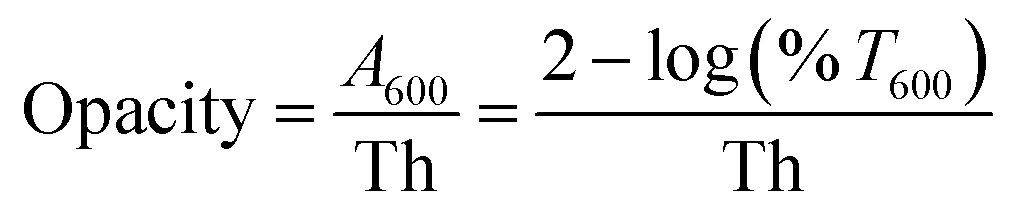
where *A*_600_ and % *T*_600_ represent the absorbance and transmittance values at 600 nm. Th (mm) is the thickness of the investigated film. Each measurement was performed in triplicate, and the results are reported as mean ± standard deviation (SD).

The UV-blocking capacity of the fabricated films was calculated using the following equation, with data processing performed in Origin software:^33^2
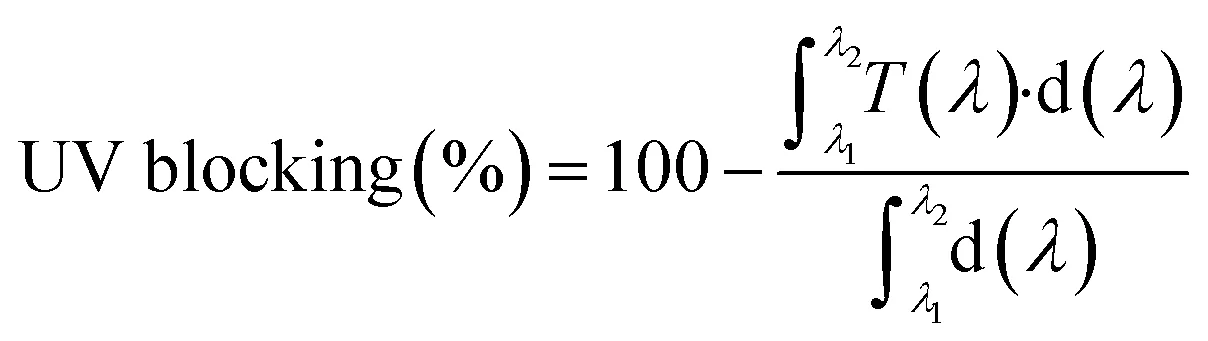
where *T*(*λ*) is the film's transmittance, and *λ*_1_ and *λ*_2_ define the wavelength range of interest, including 200–280 nm for UVC, 280–320 nm for UVB, and 320–400 nm for UVA. All measurements were conducted in triplicate, and the data are reported as mean ± standard deviation (SD).

##### 2.3.2.3 Mechanical properties

The films were cut into strips (10 × 50 mm^2^) and secured in a testing fixture with a gauge length of 24 mm for mechanical characterization. Tensile strength (TS, MPa) and elongation at break (EB, %) were determined using a texture analyzer (Brookfield CT3, TA-RCA, Ametek Brookfield, USA) at a crosshead speed of 3 mm s^−1^. All measurements were conducted in quintuplicate, and the data are reported as mean ± standard deviation (SD).

##### 2.3.2.4 Water swelling and water solubility

The films were cut into strips of 10 × 20 mm^2^ and dried in an oven at 45 °C for 24 h to achieve complete dehydration. The initial dry mass of each sample was recorded as *W*_0_. The dried films were then placed in centrifuge tubes containing 20 mL of double-distilled water and kept at room temperature for 24 h. After immersion, the samples were gently blotted with tissue paper to eliminate excess surface moisture, and their swollen weights were recorded as *W*_1_. The films were subsequently dried again at 45 °C for 24 h, and the final dry weights were recorded as *W*_2_. All measurements were performed in triplicate, and the results are presented as mean ± SD.

The water swelling was calculated using the equation:^34^3
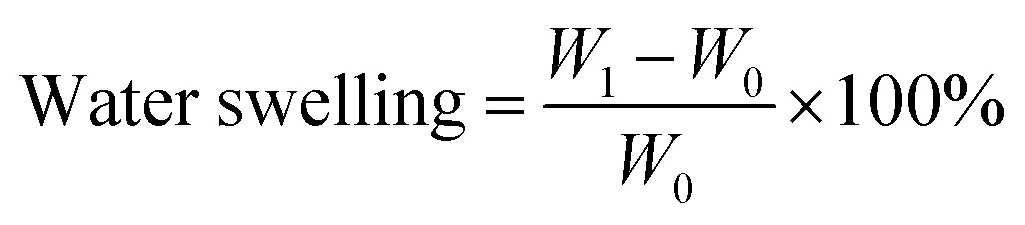


The water solubility was calculated using the equation:^35^4
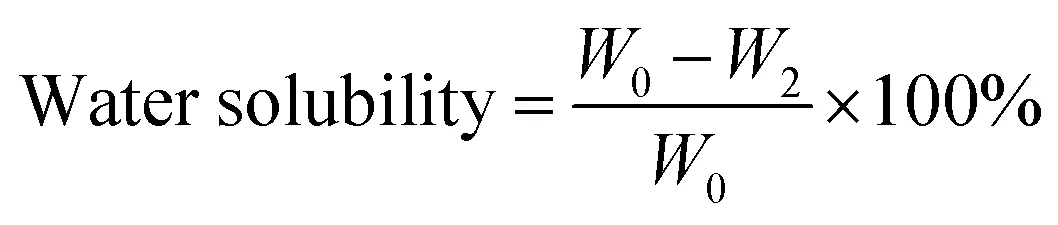


##### 2.3.2.5 Thermal stability

The thermal stability of the films was evaluated using a thermogravimetric analyzer (LINSEIS-STA PT 1600, Germany). Measurements were conducted under a nitrogen flow of 20 mL min^−1^ at a heating rate of 10 °C min^−1^ across a temperature range of 40–650 °C.

### 2.4 Antioxidant activities

The antioxidant activity of PVA/chitosan films incorporating CDs/Ag/AgCl nanocomposites was evaluated using the DPPH radical scavenging assay. A 0.1 mM DPPH solution was freshly prepared in a methanol/H_2_O mixture (2 : 1, v/v). A piece of film samples of 0.1 g or 0.2 g were immersed in 3 mL of the DPPH solution and incubated in the dark for 30 min. The absorbance of the DPPH solutions, with and without film incubation, was measured at 522.5 nm and denoted as *A* and *A*_0_, respectively. The antioxidant activity was calculated using the following equation:5
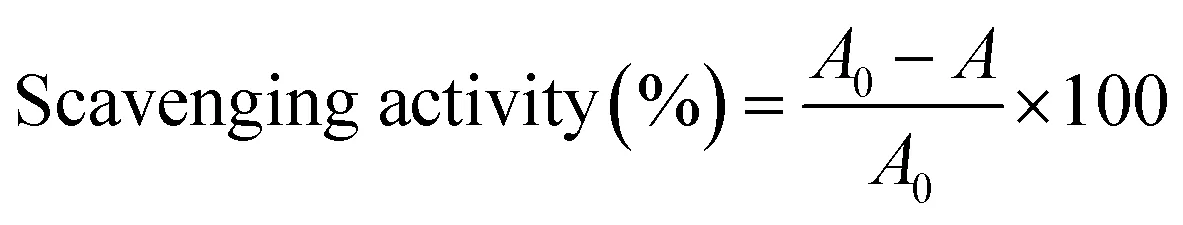


All measurements were performed in triplicate and the results are expressed as mean ± SD.

### 2.5 Antibacterial activities

*Escherichia coli* (ATCC 25913) and *Staphylococcus aureus* (ATCC 29213) were used as model organisms to evaluate the antibacterial activity of PVA/chitosan films incorporating CDs/Ag/AgCl nanocomposites using the disk diffusion method. For the assay, bacterial cultures were grown in nutrient broth at 37 °C for 16 h with gentle shaking (150 rpm), followed by serial dilution with sterile saline to obtain a final concentration of 10^8^ cfu mL^−1^. Subsequently, 100 µL of the bacterial suspension was uniformly spread onto pre-prepared nutrient agar plates. Sterile filter paper discs (6 mm in diameter) were immersed in the corresponding film-forming solutions for 1 h, dried, and placed onto the inoculated agar surfaces. The plates were then incubated at 37 °C for 24 h, and antibacterial activity was determined by measuring the diameters of the inhibition zones surrounding the discs. All measurements were repeated 4 times and the results are presented as mean ± SD.

### 2.6 UV protection application

The UV-shielding performance of PC films incorporating CDs and varying Ag contents was evaluated based on their ability to protect guava fruits from UV irradiation. Fresh guavas of uniform size and color, free of visible defects, were selected, thoroughly washed with water, and dried with tissue paper. The fruits were then placed in paper cups and covered with the respective films. A UV lamp (260 nm, 4 W) was positioned approximately 30 cm above the fruit surface, and continuously irradiated for 5 days. The guavas were photographed at 24 h intervals after removing the covering films.

### 2.7 Packaging application

Fresh strawberries of uniform size, color, and maturity, free from visible defects, were selected. The fruits were firstly rinsed with tap water, soaked in a saline solution for 5 min, and subsequently washed with double-distilled water. Residual surface moisture was removed using tissue paper, after which each fruit was individually placed in a cup covered with the corresponding films. The tape was used to position the film and ensure an airtight sealing. The visual quality of both unwrapped and wrapped strawberries was monitored during storage by capturing images at regular time intervals. All experiments were performed in triplicate.

### 2.8 Statistic analysis

All experimental data were subjected to statistical evaluation using one-way analysis of variance (ANOVA) implemented in SPSS software. Post-hoc comparisons were performed using Tukey's multiple comparison test, with statistical significance established at *p* < 0.05. Differences among groups within the same experiment were indicated by distinct letter annotations.

## 3 Results and discussion

### 3.1 Characteristics of Ag/AgCl nanocomposites

**Fig. 1:**
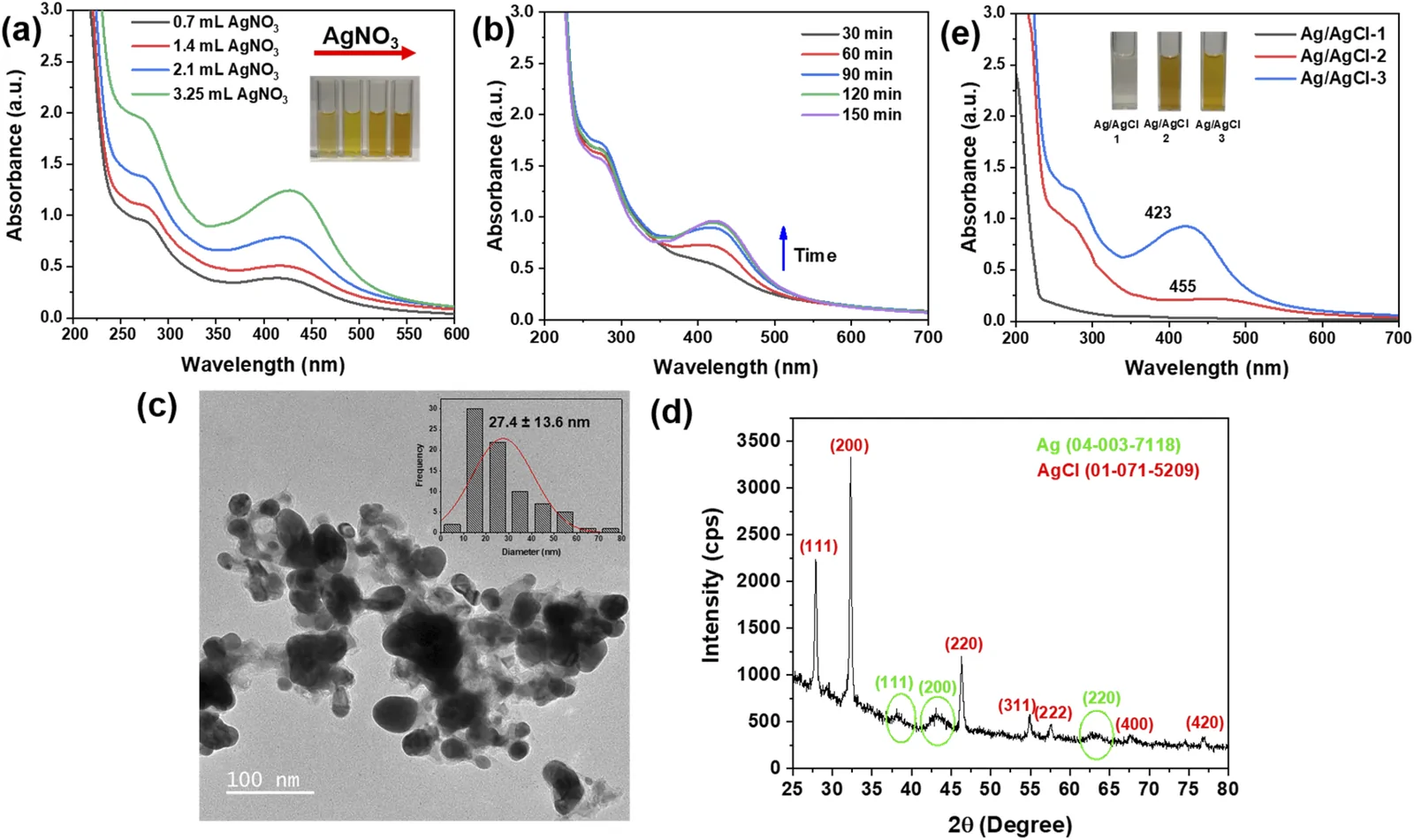
(a) UV-vis spectra of Ag/AgCl nanoparticles synthesized with varying volumes of AgNO_3_ precursor; (b) time-dependent UV-vis spectra of representative Ag/AgCl nanoparticles (2.1 mL AgNO_3_); (c) TEM image, and (d) XRD pattern of the representative Ag/AgCl nanoparticles; (e) UV-vis spectra of Ag/AgCl nanoparticles synthesized in the presence of chitosan only (Ag/AgCl-1), CDs only (Ag/AgCl-2), and both chitosan and CDs (Ag/AgCl-3).

The UV-vis spectra of the synthesized Ag/AgCl nanoparticles exhibit an absorbance peak at ∼280 nm,^36^ attributed to the formation of AgCl nanoparticles, and a peak at 425 nm, assigned to the localized surface plasmon resonance of metallic Ag nanoparticles (Fig. 1a).^23^ The peak intensity increases with increasing the volume of AgNO_3_ added to the reaction solution. Correspondingly, the color of the Ag/AgCl dispersion becomes progressively darker brown, indicating the formation of a higher concentration of nanoparticles. Time-dependent UV-vis spectra recorded at 30 min (Fig. 1b), exhibit a distinct absorption band at 280 nm and a shoulder at 425 nm, characteristics of AgCl nanoparticles.^36^ The formation of AgCl nanoparticles is attributed to the precipitation of Ag^+^ ions with Cl^−^ ions present in the CDs dispersion.^18^ With increasing reaction time, the intensity of the 425 nm band progressively increases and becomes more pronounced, reaching a maximum at 120 min, indicative of the formation of metallic Ag nanoparticles. Beyond 120 min, the absorbance reaches a plateau, suggesting that a reaction time of 120 min is sufficient for complete nanoparticle formation.

The TEM images reveal that the Ag/AgCl nanoparticles are quasi-spherical, with an average diameter of approximately 27.4 ± 13.6 nm (Fig. 1c). The XRD pattern demonstrates the coexistence of both AgCl and metallic Ag phases (Fig. 1d). Characteristic diffraction peaks at 2*θ* values of 27.8°, 32.2°, 46.3°, 54.8°, 57.6°, 67.5°, and 76.8° are indexed to the (111), (200), (220), (311), (222), (400), and (420) crystal planes of AgCl (JCPDS No. 01-071-5209).^25^ In addition, weak diffraction peaks observed at 2*θ* values of 38.3°, 43.2°, and 63.1° are assigned to the (111), (200), and (220) crystal planes of face-centered cubic (fcc) Ag (JCPDS No. 04-003-7118).^37^ The peak intensities reflect the degree of crystallinity in the synthesized Ag and AgCl nanoparticles.^37^

To elucidate the reaction mechanism, control experiments were conducted under different reaction conditions, in which Ag/AgCl nanoparticles were synthesized in the presence of chitosan only (denoted as Ag/AgCl-1), CDs only (Ag/AgCl-2), and both chitosan and CDs (Ag/AgCl-3) (Fig. 1e). In the presence of chitosan alone, no characteristic absorbance peaks corresponding to Ag or AgCl nanoparticles are observed, and the solution remains nearly transparent, indicating that chitosan cannot reduce Ag^+^ ions under these conditions. In contrast, the sample containing only CDs exhibits an absorption band at ∼280 nm and another at 455 nm, and the dispersion is characterized by a brown color, suggesting limited formation of Ag/AgCl nanoparticles due to the relatively weak reducing and stabilizing capabilities of CDs. Notably, when both CDs and chitosan are present, the absorbance peak becomes sharper and shifts to 423 nm. This blue shift indicates the formation of smaller Ag nanoparticles.^38^ In this system, chitosan acts as a stabilizing agent, preventing nanoparticle aggregation, while CDs function as reducing agents, collectively promoting the formation of smaller and more uniformly distributed Ag/AgCl nanoparticles. CDs were also used as reducing agents in the synthesis of silver nanoparticles *via* microwave-assisted and hydrothermal methods.^39,40^

### 3.2 Characteristics of PVA/chitosan film co-incorporating CDs and Ag/AgCl nanoparticles

#### 3.2.1 Optical properties

**Fig. 2:**
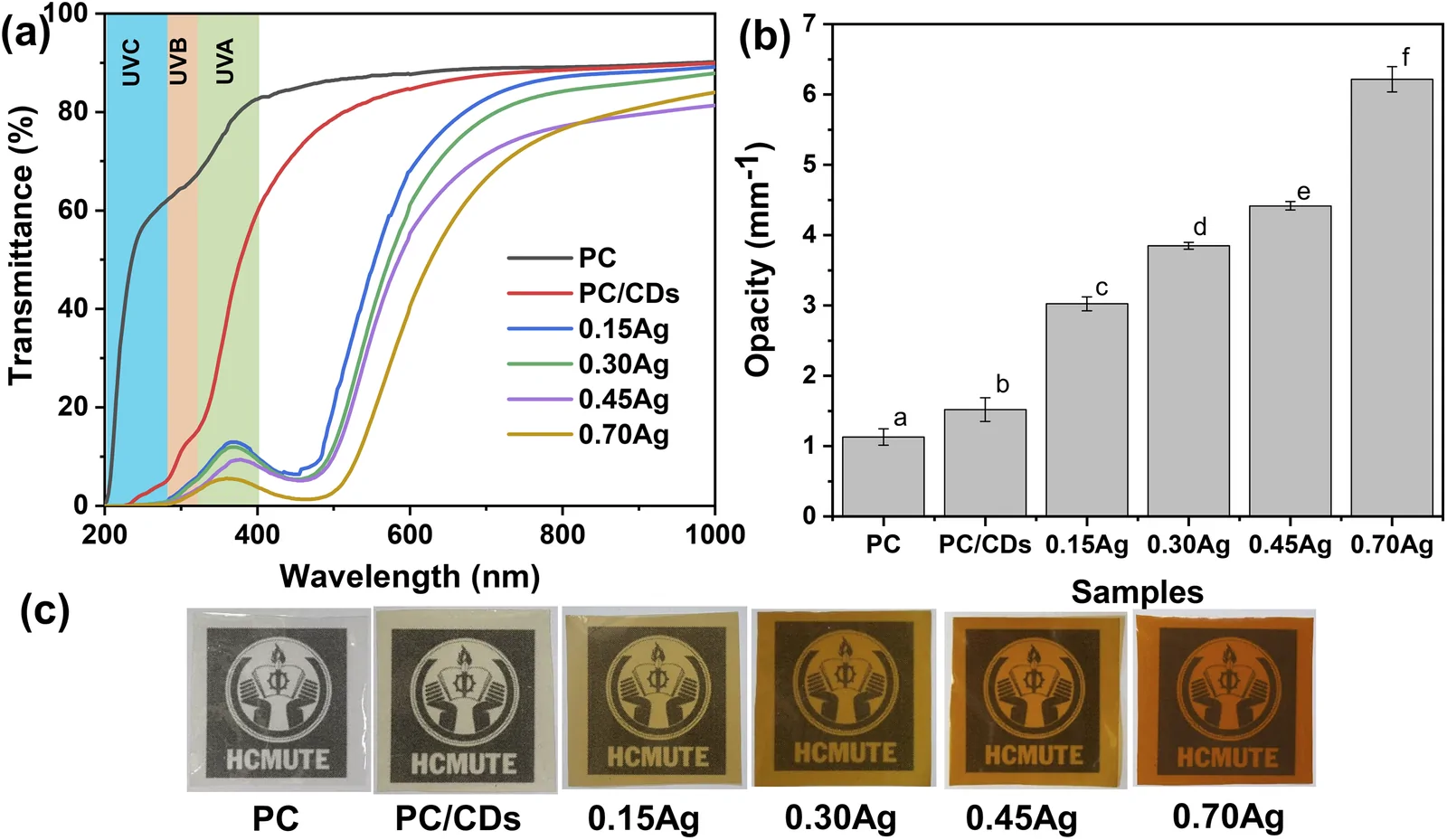
(a) Transmittance spectra, (b) calculated opacity, and (c) photographs of pristine PC films, PC/CDs films, and PC/CDs/Ag films with varying Ag contents (0.15–0.70%).

The transmittance spectra of the pristine PVA/chitosan (PC) films and PC films incorporating CDs (PC-CDs) and varying Ag contents were recorded and are presented in Fig. 2a. The pristine PC film exhibits high optical transmittance, whereas a moderate decrease is observed upon incorporation of CDs and a more pronounced reduction occurs with the addition of both CDs and Ag/AgCl nanoparticles, particularly in the UV region (200–400 nm). Accordingly, the film opacity, evaluated at 600 nm, gradually increases with the incorporation of CDs and Ag/AgCl nanoparticles (Fig. 2b). Although the films exhibit a brownish coloration due to the presence of CDs and Ag/AgCl nanoparticles, the underlying logo remains clearly visible, indicating that the incorporation of these nanofillers does not significantly impair the films' transparency (Fig. 2c).

UV-blocking capability is a critical parameter in the development of multifunctional packaging materials.^41^ The UV-blocking efficiencies of the pristine PC films and PC films incorporating broccoli leaf-derived CDs and varying Ag contents are summarized in Table 1. The pristine PC film exhibits the lowest UV-blocking efficiency across all regions, whereas the incorporation of CDs leads to a noticeable improvement, particularly in the UVC region (from 56.86% to 97.84%). The blocking efficiency is further enhanced with the addition of Ag/AgCl nanoparticles, achieving nearly complete shielding effect in both the UVB and UVC regions. Remarkably, in the UVA region, which is primarily responsible for the generation of reactive oxygen species (ROS) and consequent food degradation,^2^ the blocking efficiency increased progressively with increasing Ag contents, from 24.24% for the pristine PC film to 61.47% for the CD-incorporated film, and further to 88.31–94.80% for films containing both CDs and higher Ag loadings. Notably, at 0.45% Ag, the composite film exhibited excellent UV-shielding performance, achieving nearly 100% shielding in the UVB/UVC regions and ∼93% in the UVA region. The UV-shielding efficacy of this composite film is comparable to that of other high-performance films reported in the literature.^17,33,42,43^ Notably, compared with PVA/chitosan films incorporating Ag@CDs nanocomposites reported by Du *et al.*, the significantly higher UV-blocking efficacy of this composite film is attributed to the high loading content of CDs within the film.^27^ The enhanced UV-blocking performance is attributed to the strong UV absorption of CDs and Ag/AgCl nanoparticles, thereby highlighting their considerable potential for applications requiring effective UV protection.

**Table 1:** Calculated UV-blocking capacity of PVA/chitosan films incorporating CDs and varying Ag contents (0.15–0.70% relative to polymer mass)

Blocking (%)	PC	PC/CDs	0.15 Ag	0.30 Ag	0.45 Ag	0.70 Ag
UVA	24.24 ± 1.60^a^	61.47 ± 1.87^b^	88.31 ± 1.64^c^	90.12 ± 0.59^cd^	92.93 ± 0.86^de^	94.80 ± 0.47^e^
UVB	36.04 ± 0.97^a^	87.97 ± 1.86^b^	95.91 ± 0.94^c^	97.22 ± 0.34^c^	98.30 ± 0.50^c^	98.37 ± 0.34^c^
UVC	56.86 ± 0.53^a^	97.84 ± 0.56^b^	99.64 ± 0.18^c^	99.78 ± 0.04^c^	99.90 ± 0.03^c^	99.89 ± 0.01^c^

#### 3.2.2 FTIR spectra

**Fig. 3:**
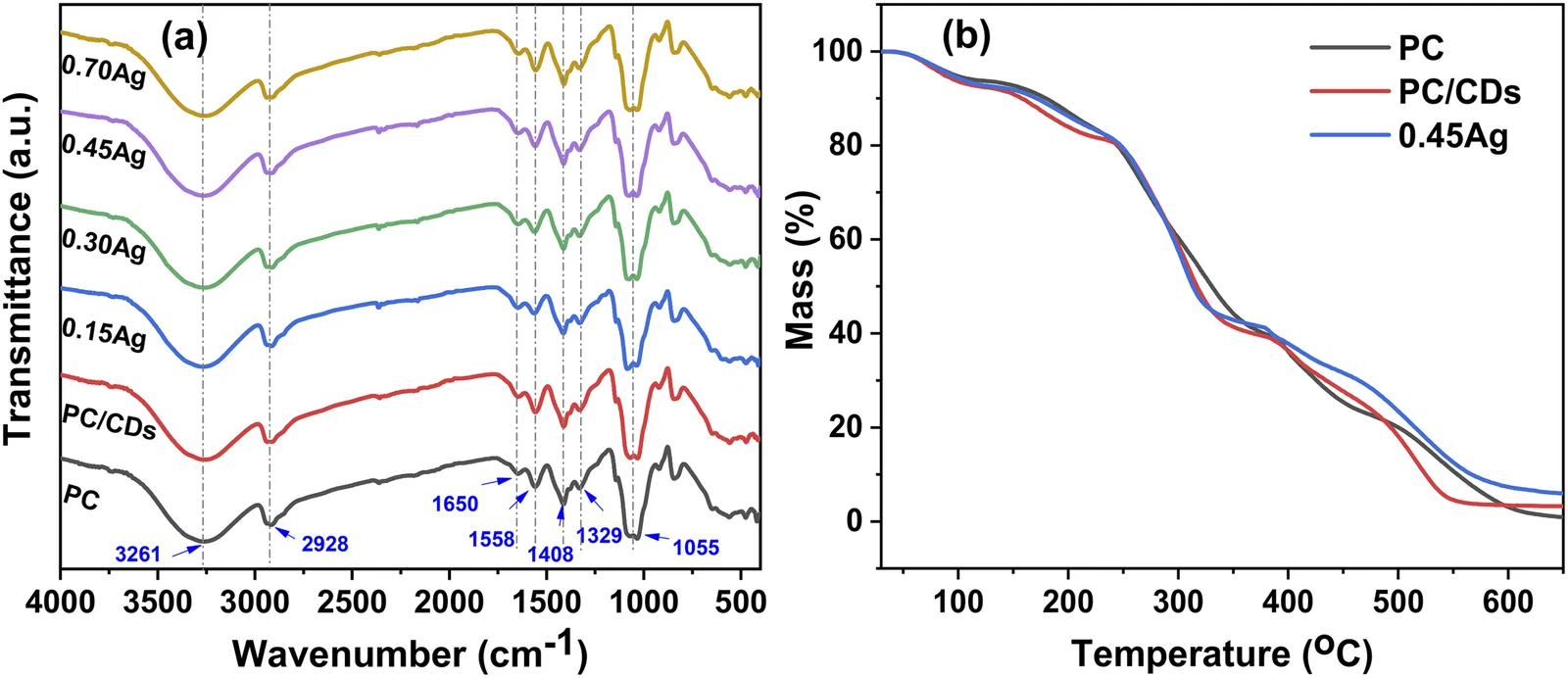
(a) FTIR spectra of PC, PC/CDs, and PC/CDs/Ag films with varying Ag contents (0.15–0.70% relative to polymer mass) and (b) TGA curves of representative films (PC, PC/CDs, and PC/CDs/0.45 Ag).

The FTIR spectra of PVA/chitosan films incorporating CDs and varying amounts of Ag/AgCl nanoparticles are presented in Fig. 3a. Overall, the spectra exhibit the characteristic absorption bands of both PVA and chitosan. Specifically, the broad band at 3261 cm^−1^ is attributed to the overlapping stretching vibrations of O–H and N–H groups. The peak at 2928 cm^−1^ is assigned to the stretching vibrations of C–H and –CH_2_ groups.^43^ The bands observed at 1650, 1558, and 1055 cm^−1^ correspond to the C=O stretching vibration of amide I, the N–H bending vibration of amide II, and the C–O stretching vibration, respectively.^44,45^ The bands at 1408 and 1329 cm^−1^ are attributed to the CH_2_ bending vibrations.^46^ Notably, the incorporation of CDs and varying amounts of Ag/AgCl nanoparticles does not lead to the appearance of new FTIR bands, indicating that no new chemical bonds are formed and suggesting a good compatibility between the fillers and the polymer matrix.

#### 3.2.3 Thermogravimetric (TG) analysis

Thermal stability is a critical factor for the large-scale fabrication of polymer films *via* thermoplastic processing in industrial applications.^3^ The TG analyses were conducted on three representative films, including pristine PC, PC/CDs, and PC incorporating CDs with 0.45% Ag content, and the results are presented in Fig. 3b. Overall, all samples exhibit continuous weight loss over the temperature range of 55–650 °C, attributed to moisture evaporation, decomposition of glycerol and polymer functional groups, and subsequent carbonization. The PC/CDs film shows slightly higher weight loss, indicating a marginal reduction in its thermal stability compared with the pristine PC film. Similarly, Murugan *et al.* reported no significant change in TG curves after incorporating tangerine peel-derived CDs into PVA/chitosan films, suggesting a minimal effect of CDs on thermal stability.^43^ However, above 380 °C, the PC/CDs/0.45 Ag film exhibits obviously reduced weight loss compared with the pristine PC and PC/CDs films. The residual mass of the PC/CDs/0.45 Ag film is 6.1%, which is higher than that of the PC/CDs (3.2%) and the pristine PC (1.0%) films, demonstrating its greater thermal stability. Yang *et al.* also reported enhanced thermal stability of PVA/chitosan films upon incorporation of Ag nanoparticles, which was attributed to the presence of thermally stable metallic nanoparticles and strengthened intermolecular interaction within the polymer matrix.^22^ An improvement in thermal stability was also observed for the PVA/chitosan films loaded with ZnO nanoparticles and tannic acid, where ZnO nanoparticles act as thermal insulators by limiting the mobility of polymer chains.^11^

#### 3.2.4 Mechanical properties

**Fig. 4:**
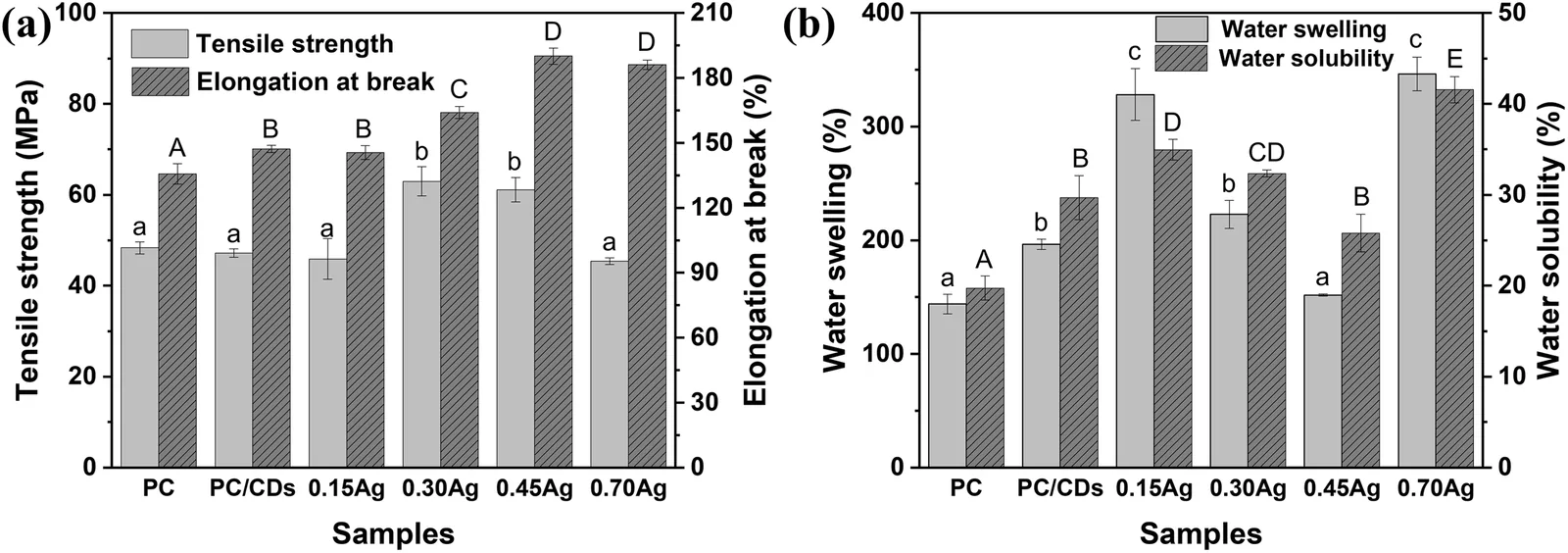
(a) Tensile strength (MPa) and elongation at break (%) and (b) water swelling and water solubility of PC, PC/CDs, and PC/CDs/Ag films with varying Ag contents (0.15–0.70%).

The mechanical properties, including tensile strength (TS) and elongation at break (EB), are crucial factors in ensuring the integrity of packaging films during preservation and transportation. The TS and EB values of the pristine PC film, PC-CDs film, and PC films co-incorporating CDs and varying Ag contents are presented in Fig. 4a. Compared to the pristine PC film, the PC/CDs film shows a negligible effect on the mechanical properties. However, the TS increases and then decreases while EB gradually increases with increasing Ag content (0.15–0.70% relative to the polymer mass). Notably, at 0.45% Ag loading, the film exhibits an optimal TS of 61.11 MPa which representing an approximately 26% improvement compared with the pristine PC film. This improvement can be attributed to the enhanced intermolecular interactions within the polymer matrix through the formation of hydrogen bonding and van der Waals forces, thereby reinforcing the film structure. In contrast, a slight reduction in TS at 0.70% Ag loading was attributed to the disruption of intermolecular interactions between the polymer network and the nanofillers caused by excessive nanoparticle loading. A similar trend was reported by Gasti *et al.*, who observed that the TS of PVA/chitosan films initially increased and subsequently decreased as the loading of *in situ*-synthesized Ag nanoparticles increased from 0.15 to 0.45 wt%.^30^ Furthermore, the PC/CDs/0.45 Ag film exhibits an increase in EB to ∼190%, representing an improvement of approximately 40% compared with the pristine PC film. The TS and EB of the PC/CDs/0.45 Ag film are comparable to, and in some cases exceed, those reported for PVA/chitosan films incorporating other nanofillers.^3,11,22,43,47^ Notably, according to the report by Du *et al.*, PVA/chitosan films incorporating Ag@CDs nanocomposites at a loading of 3 wt% exhibit a TS of 39.13 MPa and an EB of ∼100%, which are significantly lower than those obtained in this study.^27^ This may be attributed to the smaller size of the Ag/AgCl nanoparticles and the higher CDs loading in our system.

The simultaneous enhancement in both TS and EB for the PC/CDs/0.45 Ag film, an uncommon phenomenon, is attributed to strong intermolecular interactions and the uniform dispersion of nanofillers within the polymer matrix.^29^ Similarly, Abtalib *et al.* reported concurrent increases in TS and EB upon incorporation of Ag and TiO_2_ nanoparticles into a chitosan/polyethylene oxide blend, which they ascribed to increased structural order arising from intense hydrogen bonding interactions induced by the nanofillers.^48^ Consistent with these findings, SEM cross-sectional images (Fig. S1) show no discernible change in the surface morphology of the PC film after incorporating CDs and Ag/AgCl nanoparticles at 0.45% Ag, while EDX elemental mapping (Fig. S2) confirms the homogeneous dispersion and good compatibility of these nanofillers within the matrix.

#### 3.2.5 Water swelling and water solubility

Water swelling and water solubility are critical parameters for evaluating the water resistance of packaging films, particularly for applications involving high-moisture foods or storage under high-humidity conditions. The corresponding values for PC films incorporating CDs and varying amounts of Ag/AgCl nanoparticles are presented in Fig. 4b. The pristine PC film exhibits the lowest swelling degree and water solubility of 143.93% and 19.74%, respectively, reflecting a dense network formed by strong intermolecular interactions between PVA and chitosan.^49^ The incorporation of CDs increases both parameters due to their hydrophilic nature and partial disruption of polymer interactions. A further increase in these parameters is observed at low Ag loading (0.15%), indicating enhanced water uptake due to the partial loosening of the polymer network. As the Ag content increases to 0.45% (PC/CDs/0.45 Ag film), both parameters decrease to values close to those of the pristine film. This behavior indicates that, at moderate filler loading, the nanoparticles act as effective nanofillers or physical crosslinking points, leading to a more compact and structurally integrated network that restricts water penetration.^50^ However, at higher Ag content (0.70%), both swelling and solubility increase significantly. This deterioration in water resistance can be attributed to the formation of interfacial defects or microvoids (as evidenced in Fig. S1), which facilitate water diffusion and polymer chain disentanglement.

Furthermore, the water vapor transmission rate (WVTR) and contact angle, two key parameters for evaluating the water barrier properties of the films, are presented in Fig. S3 and S4. The WVTR gradually decreases with the incorporation of CDs and increasing Ag loadings from 0 to 0.45%, reaching the lowest value for the PC/CDs/0.45 Ag film, and then increases at 0.70% Ag loading. This improvement at moderate loading is attributed to the synergistic effect of CDs and Ag/AgCl nanoparticles, which act as nanofillers to fill microvoids, enhance structural compactness, and create a tortuous diffusion pathway that restricts water vapor transport.^50^ At higher loading, however, the barrier performance deteriorates due to the formation of interfacial defects and microvoids that facilitate vapor diffusion. Consistently, the contact angle increases from 83.6° for the pristine PC film to a maximum of 93.5° for the PC/CDs/0.45 Ag film, followed by a slight decrease to 91.2° as the Ag content reaches 0.70%. This trend reflects enhanced surface hydrophobicity at moderate filler loading, resulting from increased surface roughness and reduced availability of hydrophilic groups due to intermolecular interactions. At excessive loading, structural defects may disrupt surface uniformity and partially restore hydrophilic sites, leading to the observed decrease in contact angle.

Overall, these results indicate that the PC/CDs/0.45 Ag film achieves an optimal balance between network densification and structural integrity, resulting in enhanced water resistance and barrier performance.

#### 3.2.6 Free radical scavenging capability

**Fig. 5:**
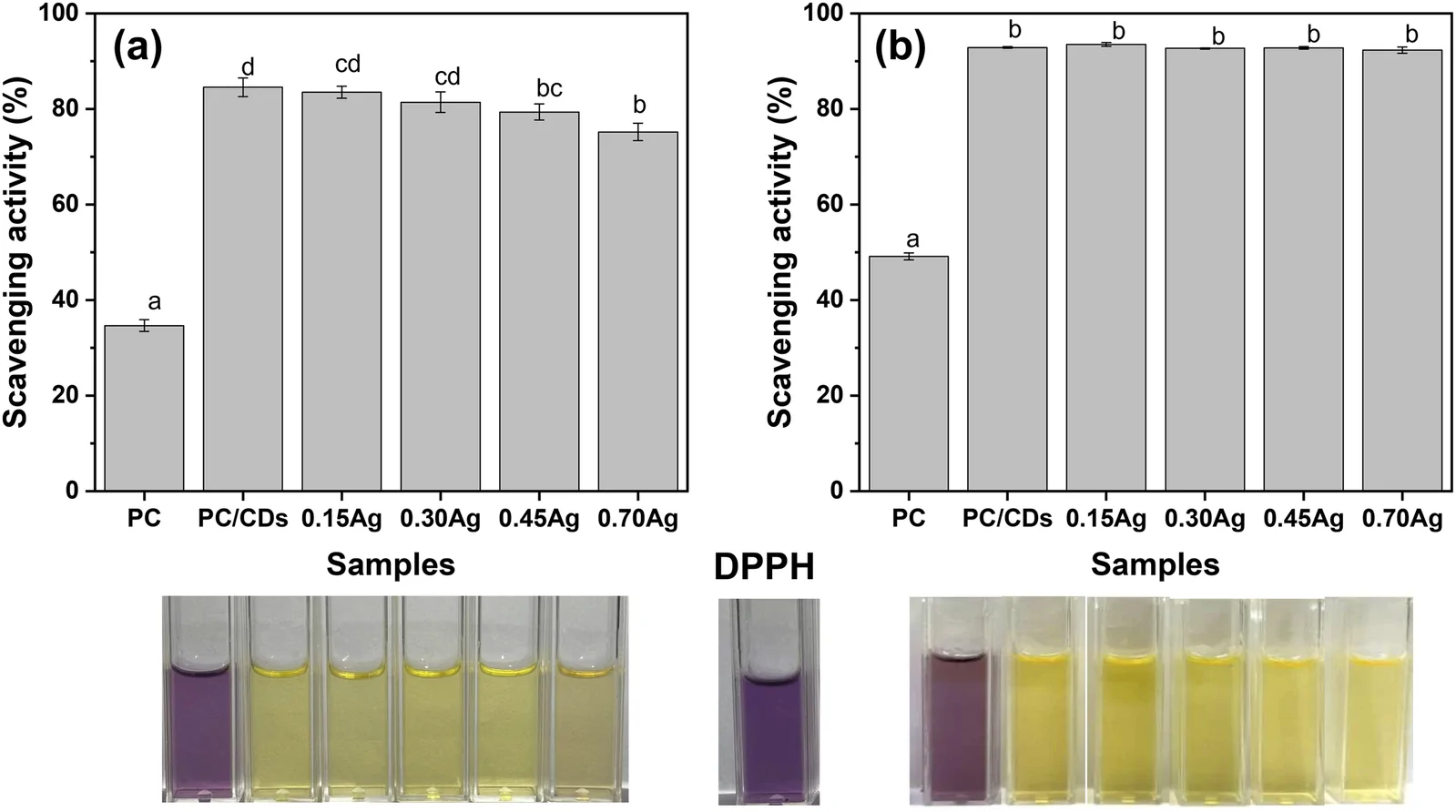
DPPH scavenging activity of (a) 0.1 g and (b) 0.2 g films (PC, PC/CDs, and PC/CDs/Ag, 0.15–0.70 wt% Ag) after reaction with DPPH solution, with corresponding photographs.

To develop multifunctional packaging films, the incorporation of antioxidant activity is of significant interest, particularly for highly perishable foods that are susceptible to oxidative degradation induced by free radicals. The free radical scavenging activity of polymer films incorporating CDs and varying amounts of Ag/AgCl nanoparticles is presented in Fig. 5. DPPH, a stable free radical characterized by a deep purple color and a characteristic absorbance peak at 522.5 nm, was employed to evaluate the antioxidant activity. Upon interaction with electron- or hydrogen-donating species, DPPH is reduced to DPPH-H, accompanied by a color change from purple to yellow. At a film dosage of 0.1 g, the pristine PVA/chitosan film exhibits a moderate radical scavenging activity of approximately 34.7%, which can be attributed to the presence of amine groups in its chemical structure.^49^ Upon incorporation of CDs, the scavenging activity increases significantly to 84.6%, demonstrating the strong antioxidant capability of the loaded CDs. This enhancement is consistent with previous studies, where broccoli leaf-derived CDs exhibited excellent antioxidant activity due to the presence of abundant functional groups and heteroatoms including N and S in their structure.^18^ Interestingly, the antioxidant activity of the films gradually decreases to 75.2% with increasing Ag loadings to 0.70%. Correspondingly, the DPPH solution exhibits a less intense yellow color at higher Ag loadings. This phenomenon can be attributed to the consumption of electron-donating functional groups on CDs during the reduction of Ag^+^ to metallic Ag, which reduces the availability of active sites responsible for radical scavenging. In addition, the negligible contribution of Ag/AgCl nanoparticles to the DPPH radical-scavenging activity can be attributed to their embedment within the polymer matrix during the *in situ* synthesis process, which hinders direct contact between the Ag/AgCl nanoparticles and DPPH radicals. In contrast, the enhanced radical-scavenging activity of *in situ*-synthesized Ag nanoparticles using fruit/plant extracts as reducing agents reported by Gasti *et al.* and Hunashyal *et al.* was attributed to the use of film-forming solutions rather than cast films during the DPPH assay, allowing more effective contact between the Ag nanoparticles and DPPH radicals.^30,31^ Nevertheless, at a higher film dosage of 0.2 g, all composite films exhibit plateau scavenging efficiencies of ∼93%, which are comparable to the scavenging effect of ascorbic acid (∼93.5%) and significantly outperform those of PVA/chitosan films loaded with other nanofillers reported in the literature.^3,46,47,49^

#### 3.2.7 Antibacterial activities

**Fig. 6:**
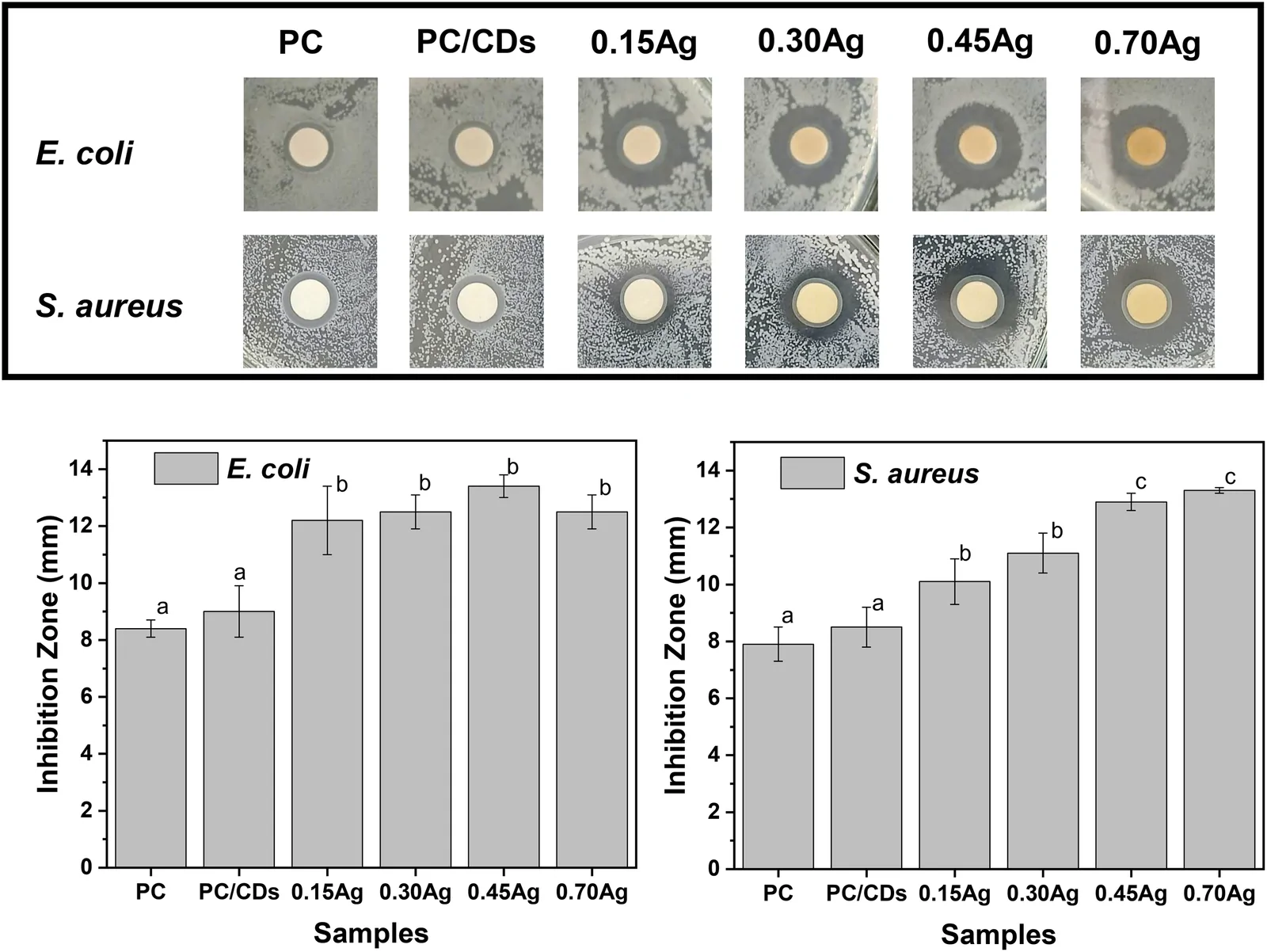
Inhibition zones of PC, PC/CDs, and PC/CDs/Ag films with varying Ag contents (0.15–0.70%) against *E. coli* and *S. aureus*, along with the corresponding plots.

The antibacterial properties of PC films incorporated with CDs and varying amounts of Ag/AgCl nanoparticles were evaluated against Gram-negative (*E. coli*) and Gram-positive (*S. aureus*) bacteria (Fig. 6), using the disc diffusion method. The pristine PC film exhibits moderate antibacterial activity, with inhibition zones of 8.4 mm for *E. coli* and 7.9 mm for *S. aureus*. This is attributed to the intrinsic antimicrobial nature of chitosan, arising from electrostatic interactions between protonated amino groups and bacterial cell membranes.^51^ Incorporation of CDs results in a slight enhancement of antibacterial activity, with inhibition zones of 9.0 mm and 8.5 mm against *E. coli* and *S. aureus*, respectively. This enhancement is attributed to the abundant surface functional groups and heteroatom doping (N and S) in CDs, which facilitate interactions with bacterial cells and induce oxidative stress, resulting in cellular damage, as reported in the literature.^17,18^ A more pronounced antibacterial effect was observed with increasing Ag contents incorporated into the polymer films. The interaction between bacteria and incorporated Ag/AgCl nanoparticles facilitates the release of Ag^+^ ions, which subsequently interact with proteins and DNA, while also promoting the generation of reactive oxygen species (ROS) that disrupt cellular functions and metabolic processes, ultimately leading to bacterial cell death.^25^ At 0.45% Ag loading, the PC/CDs/0.45 Ag film exhibits inhibition zones of 13.4 mm and 12.9 mm against *E. coli* and *S. aureus*, respectively, which are comparable to, and in some cases exceed, those reported for PVA/chitosan films loaded with *Ginkgo biloba* leaf extract,^47^ ZnO nanoparticles,^49,52^*in situ*-synthesized Cu nanoparticles,^53^ Ag nanoparticles,^22,54^ and Ag@CDs at a 3 wt% loading.^27^ Notably, PVA/chitosan films containing *in situ* synthesized Ag nanoparticles derived from *Lantana camara* leaf extract exhibited inhibition zones of 12.0 mm and 12.8 mm against *E. coli* and *S. aureus*, respectively.^31^ Although larger inhibition zones of 21 mm and 20 mm against *E. coli* and *S. aureus*, respectively, were reported for PVA/chitosan films incorporating *in situ* synthesized Ag nanoparticles using *Spondias pinnata* fruit extract, this enhancement is likely attributable to the use of low-molecular-weight chitosan, as the pristine PVA/chitosan film already exhibited inhibition zones of 17 mm and 18 mm, respectively.^30^ Furthermore, Ragab *et al.* reported even larger inhibition zones of 16.27 mm against *E. coli* and 18.49 mm against *S. aureus* for PVA/chitosan films containing biosynthesized Ag nanoparticles, which was attributed to the substantially higher Ag nanoparticle loading (3.0 wt%).^55^ The higher susceptibility of *E. coli* compared to *S. aureus* can be attributed to differences in their cell envelope structures. Specifically, *E. coli* has a thinner peptidoglycan and a more negatively-charged outer membrane, facilitating stronger interactions with positively-charged antimicrobial species, including chitosan, CDs, and Ag^+^ ions.^22,49^

Based on the above analyses, the PC/CDs/0.45 Ag film exhibits optimal mechanical properties, water resistance, UV-blocking, antioxidant, and antibacterial properties. Therefore, the PC/CDs/0.45 Ag film was selected for further investigation on practical packaging applications.

### 3.3 Preservation of guavas from UV irradiation

**Fig. 7:**
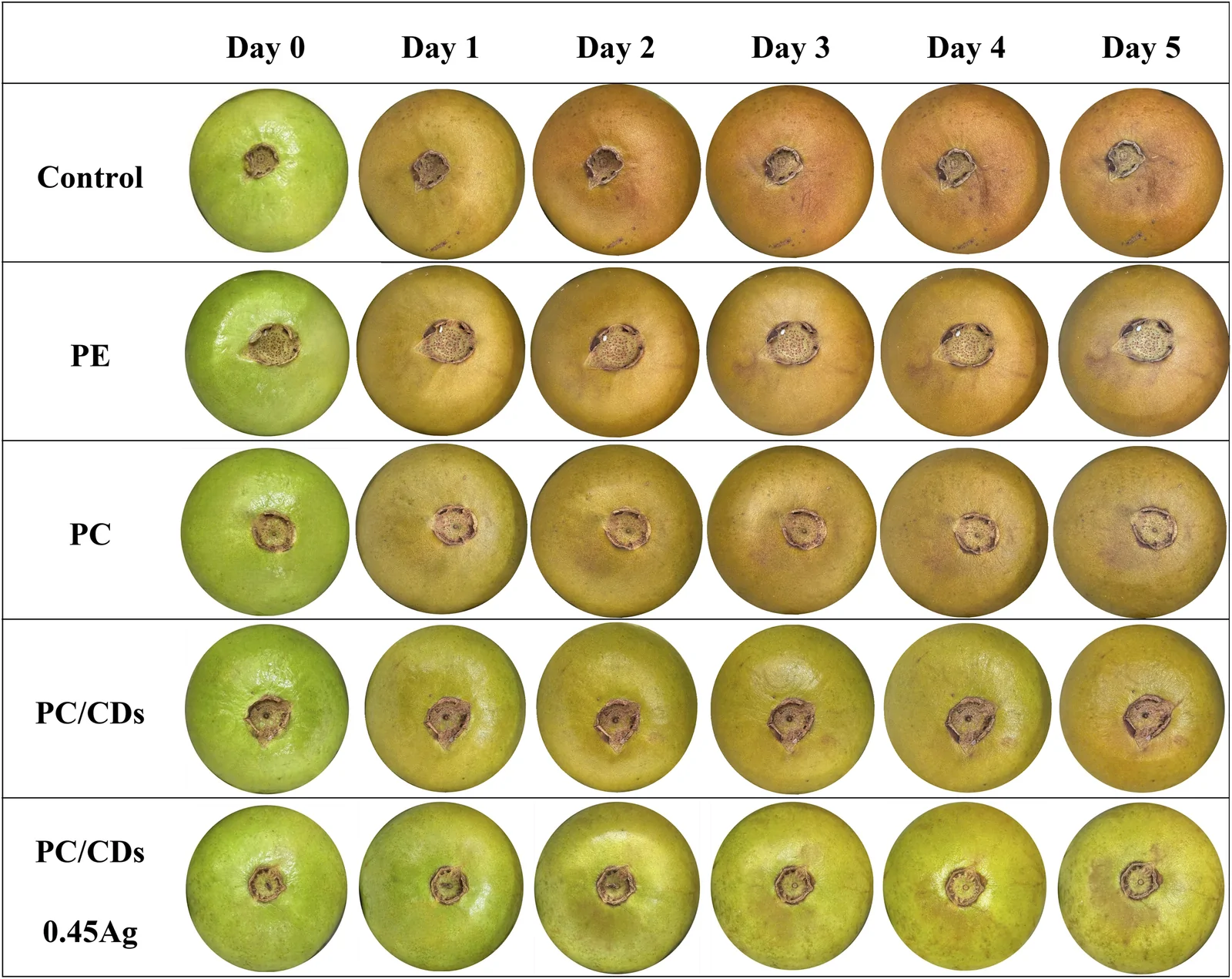
Time-dependent images of unwrapped (control) and film-wrapped guavas under UV irradiation.

UV radiation plays a critical role in food quality deterioration by triggering various spoilage reactions, including the degradation of antioxidants and proteins, lipid oxidation, the development of off-flavors, and the loss of pigments.^2^ Photographs of green guava exposed to UV irradiation at different time intervals are presented in Fig. 7. The color change (Δ*E*) was quantified using the L*, a*, b* color space and is presented in Fig. S5. As observed, unwrapped guava and those packaged with commercial PE and pristine PC films exhibit pronounced browning, indicating significant UV-induced damage. In contrast, guava packaged with PC/CDs films shows reduced discoloration due to the UV-absorbing capability of CDs. Notably, the hybrid film containing both CDs and Ag/AgCl nanoparticles provides the most effective UV protection, as evidenced by minimal color change after 5 days of storage. This enhanced performance is attributed to the synergistic UV-blocking effect of the combined nanofillers. Such a hybrid system represents an effective strategy for developing UV-resistant food packaging materials.

### 3.4 Preservation of strawberries

**Fig. 8:**
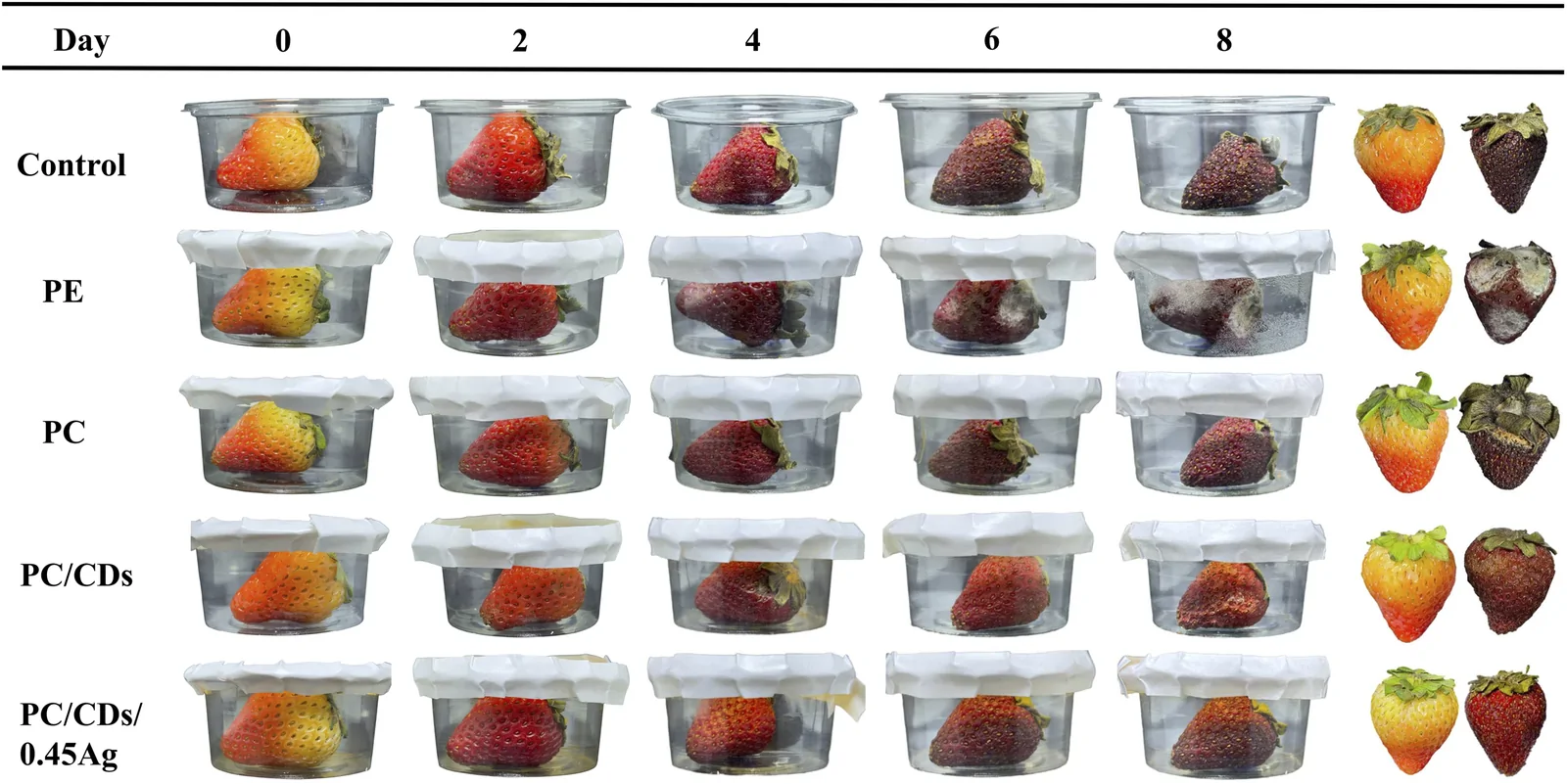
Time-dependent images of unpackaged (control) and film-packaged strawberries during storage.

Strawberries are highly nutritious yet highly perishable fruits that are particularly susceptible to microbial contamination. Images of unpackaged strawberries and those packaged with different types of films were recorded and are presented in Fig. 8. The unpackaged strawberries exhibit severe deterioration, characterized by darkening (blackening) and shrinkage due to significant moisture loss. Pronounced signs of microbial spoilage are also observed in strawberries packaged with commercial PE films, whereas less severe deterioration is noted for those packaged with pristine PC films, attributable to the intrinsic antibacterial activity of chitosan. In contrast, strawberries packaged with PC/CDs films show no visible signs of microbial contamination, owing to the inherent bactericidal activity of CDs and chitosan; however, noticeable dehydration still occurs, leading to fruit shrinkage. Notably, strawberries packaged with PC/CDs/0.45 Ag hybrid films exhibit the best preservation performance, maintaining their color and freshness and showing no signs of microbial spoilage. This performance can be attributed to the strong antibacterial activity arising from the synergistic combination of chitosan, CDs, and Ag/AgCl nanoparticles. Moreover, the compact and dense structure resulting from the filler effect of CDs and Ag/AgCl nanoparticles acts as an effective barrier to water vapor and gases, thereby reducing moisture loss and slowing the ripening process. The calculated weight loss percentage (Fig. S6) further demonstrates that strawberries packaged with the PC/CDs/0.45 Ag film exhibit lower weight loss compared to other films. Overall, these results highlight the excellent preservation capability of the PC/CDs/0.45 Ag hybrid film as a multifunctional packaging material.

According to the European Food Safety Authority (EFSA), the safety threshold for silver migration in food-contact materials is approximately 50 µg kg^−1^ food. For instance, M. El Mouzahim *et al.* reported that chitosan films containing 10 wt% AgNPs exhibited silver release levels below the EFSA safety threshold of 50 µg kg^−1^ food.^56^ Similarly, Jun Cheng *et al.* demonstrated that poly(lactic acid) (PLA)-based films incorporating 1.0 wt% AgNPs showed silver migration levels below the specific migration limit of 10 µg kg^−1^ established by Regulation (EU) No. 10/2011.^57^ A limitation of the present study is the absence of comprehensive experimental investigations on the migration behavior of silver nanoparticles from the developed nanocomposite food-packaging systems under different food simulant conditions. Nevertheless, the Ag content used in this work was relatively low (0.15–0.70%) compared with previously reported AgNP-loaded antimicrobial films.^19,26,58^ In addition, the optimized PC/CDs/0.45 Ag film exhibited low water solubility (<26%). Collectively, these findings suggest that silver migration from the developed films is likely to remain at a low level.

## 4 Conclusion

In this study, multifunctional hybrid films were successfully fabricated by simultaneously incorporating broccoli leaf-derived N,S-doped CDs and *in situ* synthesized Ag/AgCl nanoparticles into a PVA/chitosan matrix. Owing to the abundant surface functional groups, CDs acted as reducing agents while chitosan served as a stabilizing matrix, yielding quasi-spherical Ag/AgCl nanoparticles (∼27.4 nm) with mixed crystalline phases. The composite films exhibited tunable mechanical, barrier, antioxidant, UV-shielding, and antibacterial properties as a function of Ag contents (0–0.70% relative to polymer mass). With 0.45% loading, the PC/CDs/0.45 Ag film showed optimal performance, with enhanced mechanical properties (TS of 61.1 MPa, EB of 190%), improved water resistance and barrier properties, and strong UV-shielding (∼93% UVA, ∼100% UVB/UVC blocking). The films also demonstrated great antioxidant activity (∼93% DPPH scavenging), mainly attributed to CDs. Antibacterial activity against *E. coli* and *S. aureus* increased with Ag contents, reaching inhibition zones of 13.4 and 12.9 mm, respectively, for the PC/CDs/0.45 Ag film, primarily attributed to Ag/AgCl nanoparticles with synergistic contributions from chitosan and CDs. Importantly, practical evaluations confirmed that the PC/CDs/0.45 Ag film effectively protected guavas from UV-induced damage and significantly extended the shelf life of strawberries beyond that achieved with commercial PE cling film. Overall, the *in situ* synthesis strategy and eco-friendly components highlight these composite films as promising sustainable and high-performance packaging materials. The hybrid integration of active nanofillers also provides an effective strategy for designing advanced food packaging systems.

## Author contributions

Nhung Thi Tran: conceptualization, methodology, data analysis, writing – original draft, writing – review & editing, supervision, funding acquisition. Le Minh Nguyen: conceptualization, methodology, investigation, data curation, writing – original draft, writing – review & editing. Huynh Anh Le: conceptualization, methodology, investigation, data curation, writing – original draft, writing – review & editing. Thanh Nhan Le: conceptualization, methodology, investigation, data curation, writing – original draft, writing – review & editing.

## Conflicts of interest

The authors declare no conflicts of interest.

## Supplementary Material

RA-016-D6RA04270H-s001

## Data Availability

All data supporting the findings in this study is included in the article and the supplementary information (SI) files. Supplementary information is available. See DOI: https://doi.org/10.1039/d6ra04270h.
