# Supplementary material for: Incorporation of broccoli leaf-derived CDs and Ag/AgCl nanoparticles into PVA/chitosan as multifunctional food packaging films
Source: RSC Adv. 2026 Jul 29;16(39):43659–71. doi: 10.1039/d6ra04270h (PMC13417093; doi:10.1039/d6ra04270h)
Supplement: Supplementary file 1 [file RA-016-D6RA04270H-s001.pdf]

Supplementary Information (SI) for RSC Advances.
This journal is © The Royal Society of Chemistry 2026

Supporting information

Incorporation of Broccoli leaf-derived CDs and Ag/AgCl Nanoparticles into PVA/Chitosan as Multifunctional Food Packaging Film

Nhung Thi Tran[1*], Le Minh Nguyen[1], Huynh-Anh Le[1], Thanh-Nhan Le[1]

[1]Ho Chi Minh City University of Technology and Engineering, 01 Vo Van Ngan Street, Thu Duc Ward, Ho Chi Minh City 700000, Vietnam

Corresponding author: Nhung Thi Tran, email: nhungtt@hcmute.edu.vn

Note: All authors contributed equally to this work and share co-first authorship.

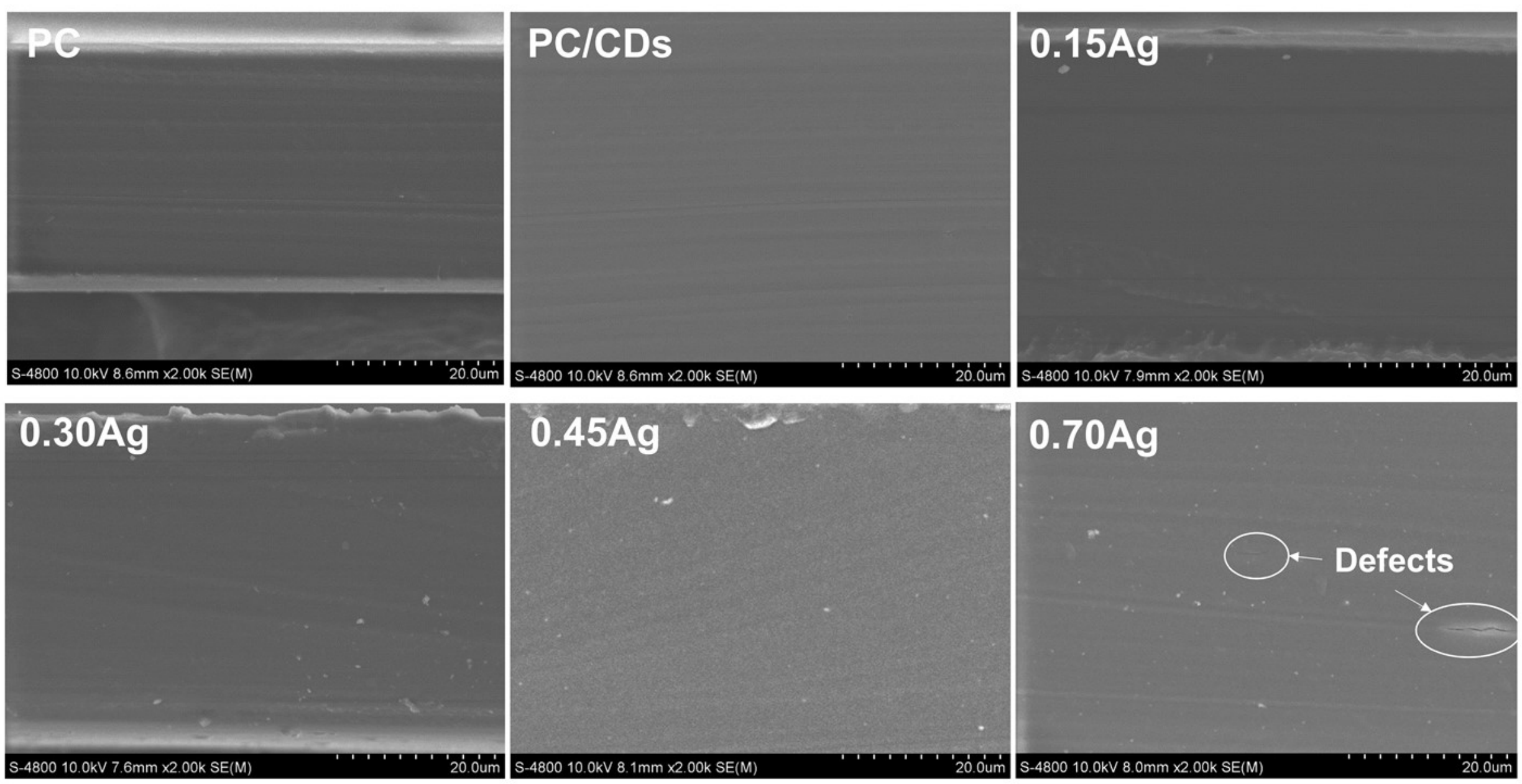

Figure S1. Cross-sectional SEM images of the pristine PVA/chitosan (PC) film, PC/CDs film, and PC films co-incorporated with CDs and varying Ag contents (0.15–0.70%, relative to the total mass of PVA and chitosan)

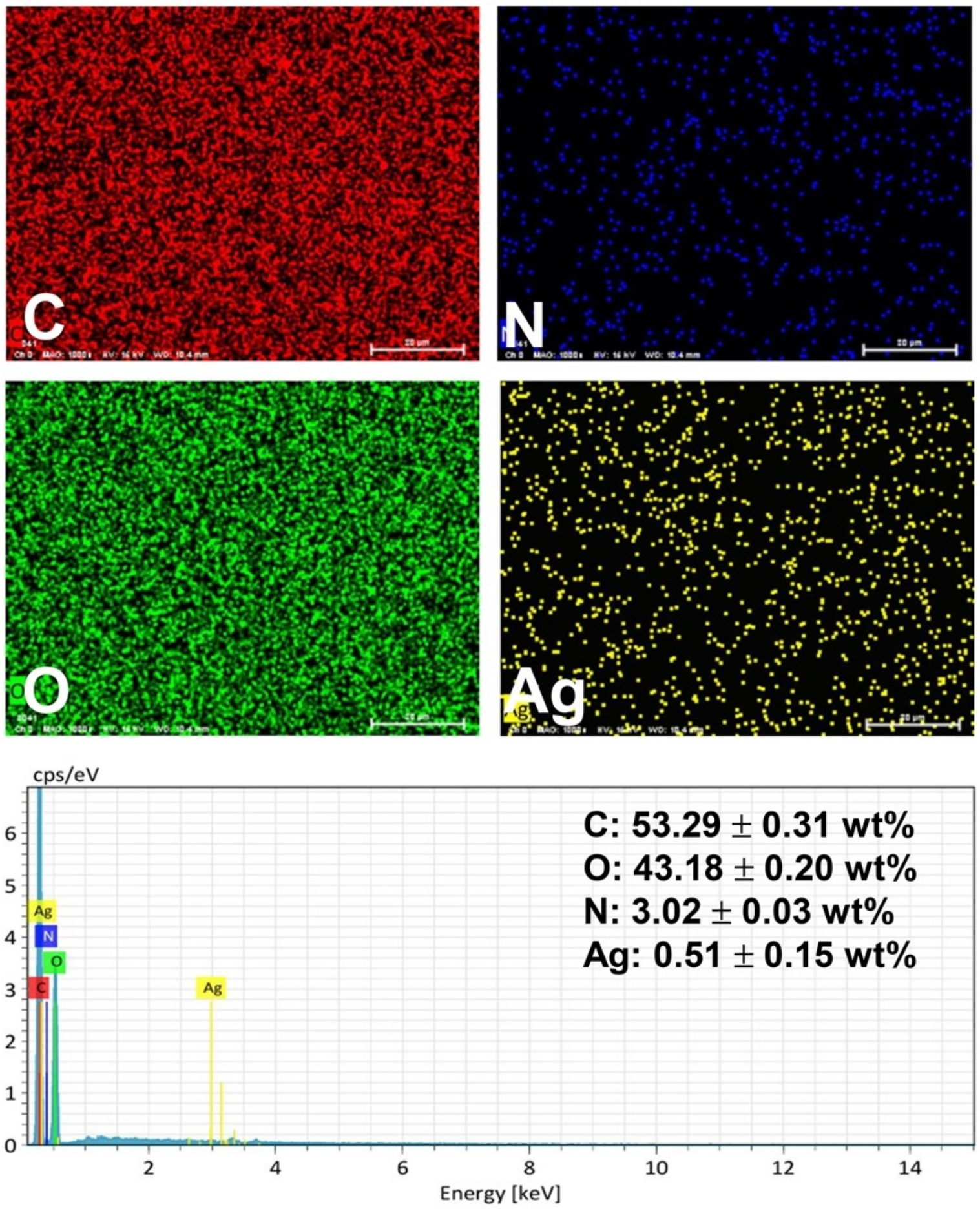

Figure S2. EDX survey and elemental mapping of the PC/CDs/0.45Ag composite film

Evaluation of water vapor transmission rate (WVTR)

The WVTR of the film samples was measured according to a previously reported method with slight modification.[1] Briefly, 10 mL of double-distilled water was placed into glass vials with an opening diameter of approximately 2 cm. The vial openings were tightly covered with the test films and sealed using Teflon tape to ensure an airtight system. The initial weight of each assembly was recorded as W_0. The vials were subsequently maintained in an oven at 45 °C for 20 h, after which the final weight (W_1) was determined. The elevated temperature used in this study (rather than 38 °C used in the standard procedure) is to accelerate water evaporation. The WVTR was then calculated using the following equation:

$$WVTR = \frac{W_o - W_1}{A \times 20} \frac{g}{(m^2 \times h)}$$

Where A is the area of permeation opening (m^2). This study is to evaluate the difference in water barrier among different film samples.

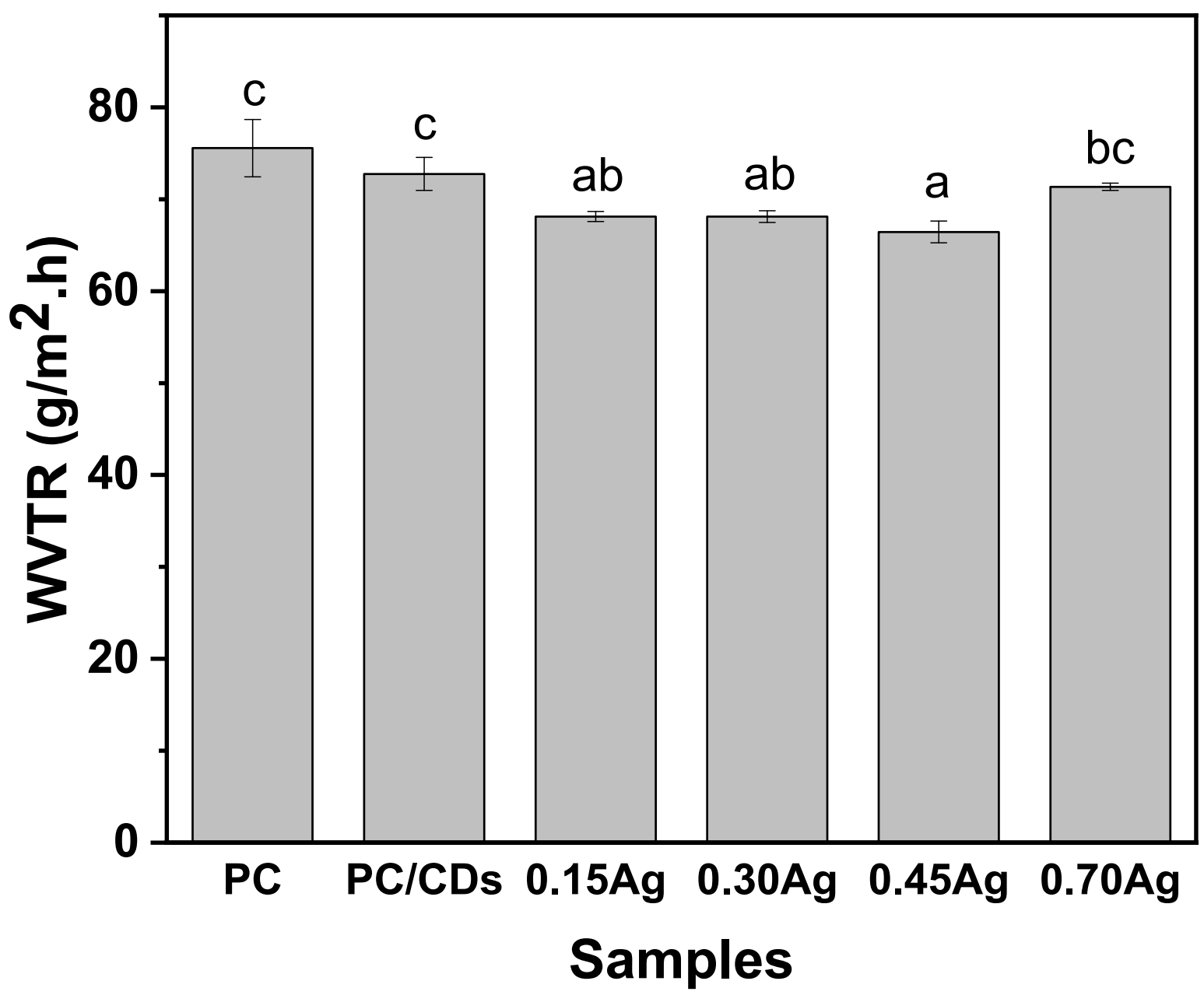

Figure S3. Water vapor transmission rate of the pristine PC film, PC/CDs film, and PC films co-incorporated with CDs and varying Ag contents (0.15–0.70%, relative to the total mass of PVA and chitosan)

Evaluation of contact angle

The contact angle of the fabricated films was measured following previously reported methods with modifications.[37, 38] Briefly, the films were cut into dimensions of 1 cm × 5 cm and placed on a flat glass substrate. A 10 μL glycerol droplet was deposited onto the film surface at three different locations using a microsyringe. The droplet profiles were captured using a digital camera, and the contact angles were subsequently determined by analyzing the droplet contours with ImageJ software. All measurements were performed in triplicate, and the results are expressed as mean ± standard deviation (SD).

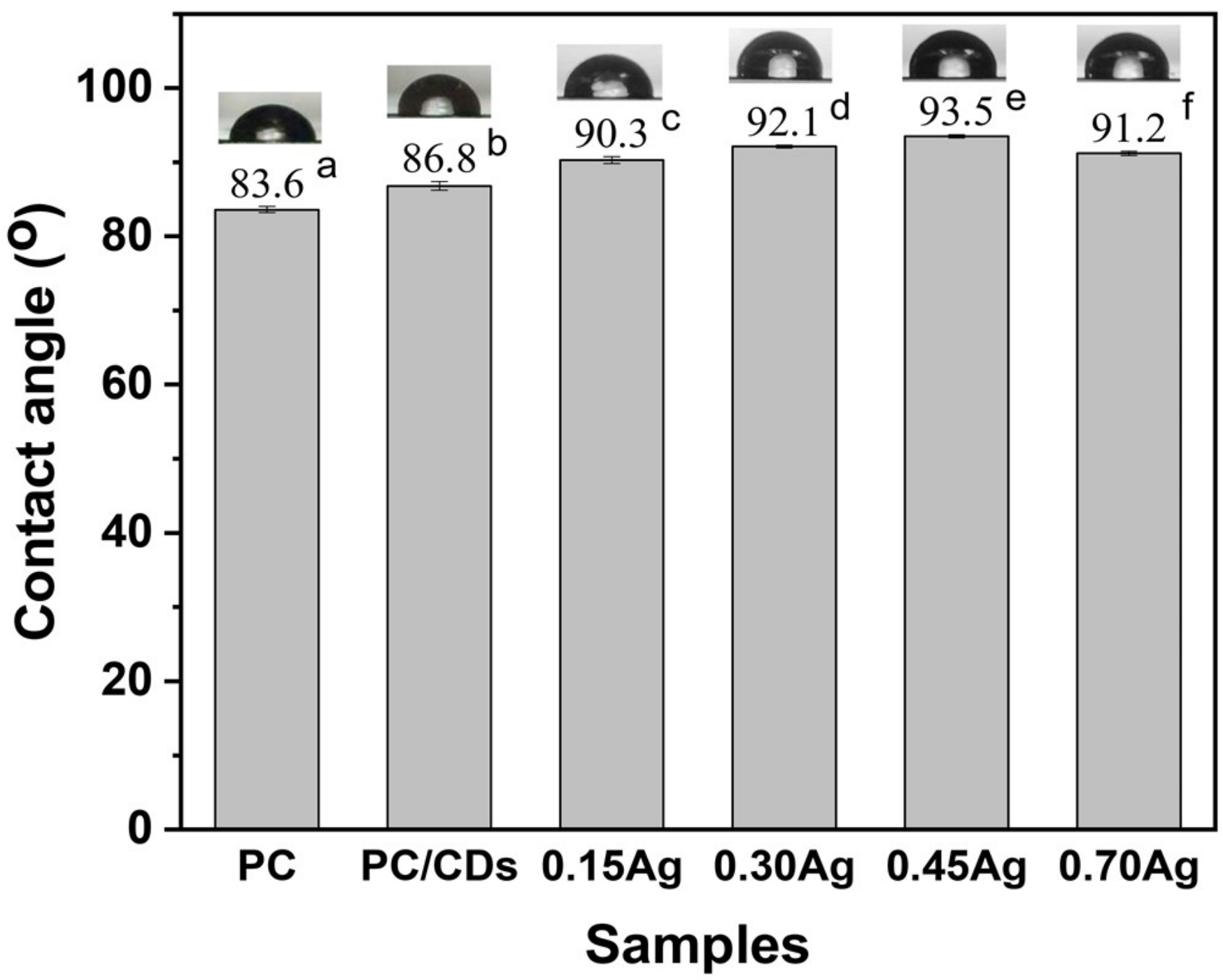

Figure S4. Contact angle of the pristine PC film, PC/CDs film, and PC films co-incorporated with CDs and varying Ag contents (0.15–0.70%, relative to the total mass of PVA and chitosan)

Evaluation of color change in guava under UV irradiation

The color of green guava under UV irradiation was monitored over time using a Chroma Meter (Konica Minolta, Inc., Japan). The overall color change (ΔE) of both film-packaged and unpackaged samples was calculated using the following equation:

$$\Delta E = \sqrt{(L^* - L_0)^2 + (a^* - a_0)^2 + (b^* - b_0)^2}$$

where L^*, a^*, and b^* represent the lightness and chromaticity coordinates of the samples at a given time, while L_0, a_0, and b_0 correspond to the initial values measured on day 0.

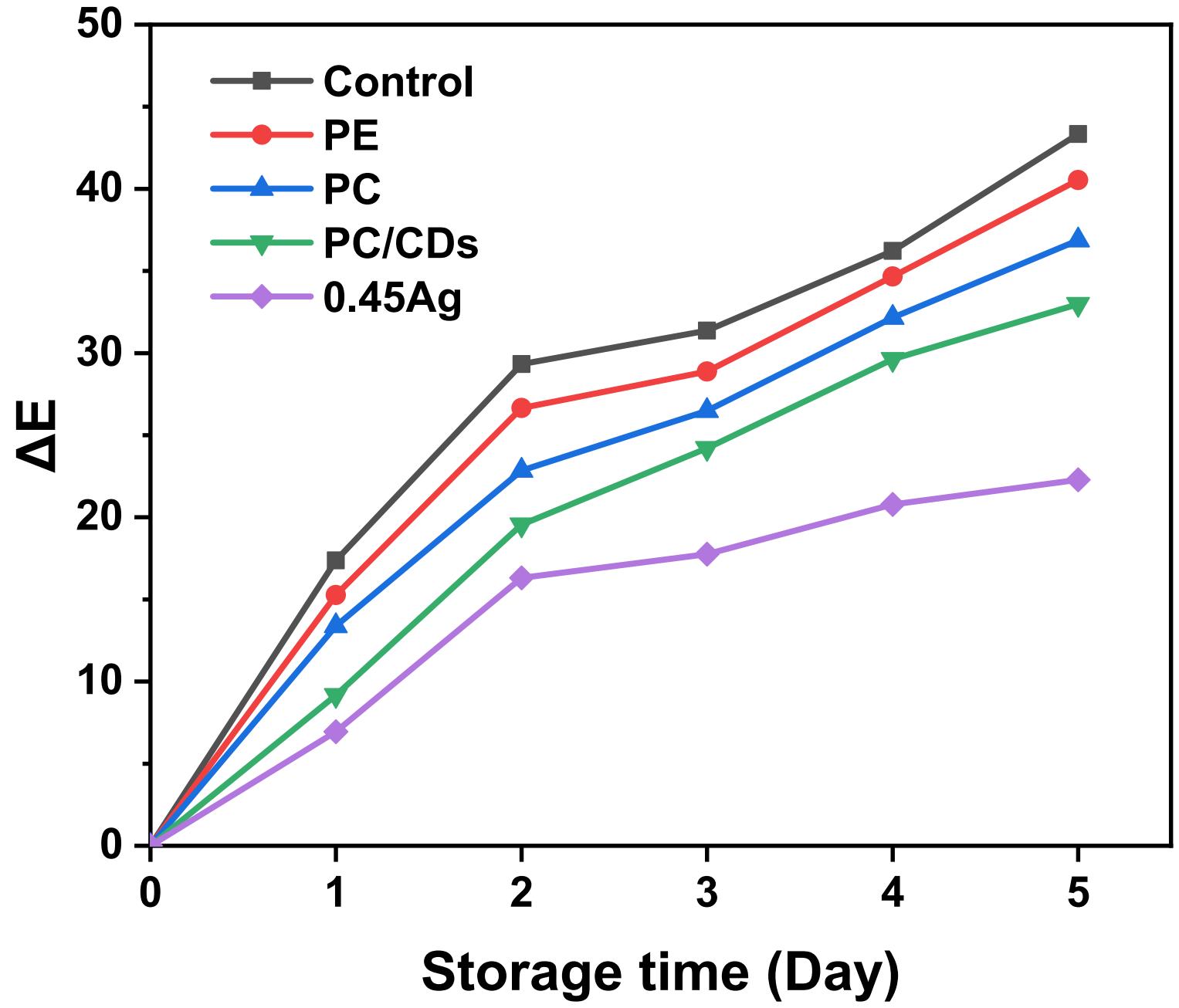

Figure S5. Color change (ΔE) of unwrapped and film-wrapped green guavas under UV irradiation.

Evaluation of weight loss in strawberries during storage

The weight loss percentages of the strawberries were calculated using the following equation:

$$Weight\ loss\ (\%) = \frac{m_o - m_t}{m_o} \times 100\%$$

Where m_o and m_t represent the weight of the investigated strawberries on day 0 and day t, respectively.

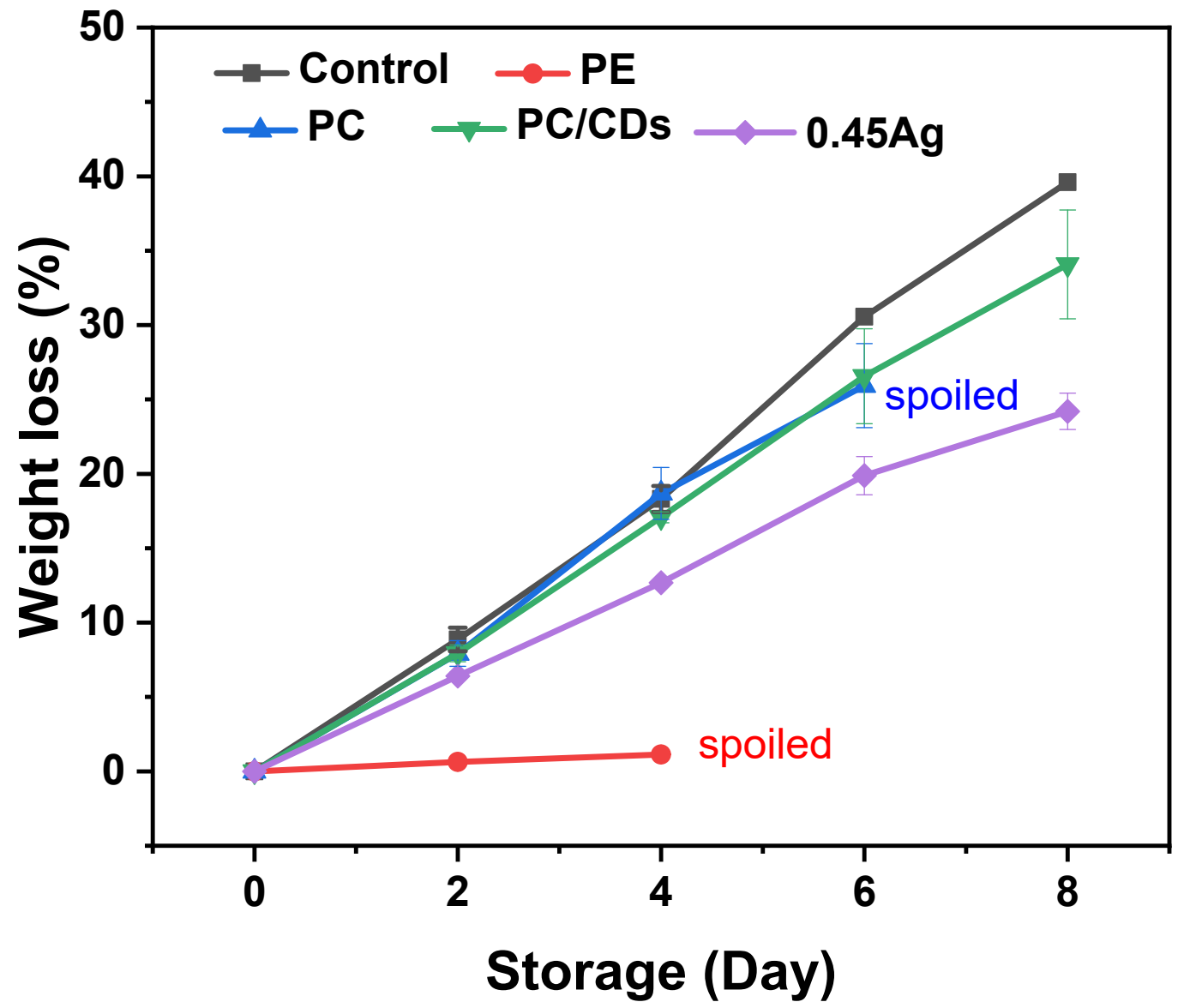

Figure S6. Weight loss (%) of unpackaged and film-packaged strawberries during storage
